# *PAUPAR* and PAX6 sequentially regulate human embryonic stem cell cortical differentiation

**DOI:** 10.1093/nar/gkab030

**Published:** 2021-02-05

**Authors:** Yanxin Xu, Jiajie Xi, Guiying Wang, Zhenming Guo, Qiaoyi Sun, Chenqi Lu, Li Ma, Yukang Wu, Wenwen Jia, Songcheng Zhu, Xudong Guo, Shan Bian, Jiuhong Kang

**Affiliations:** Clinical and Translational Research Center of Shanghai First Maternity and Infant Hospital, Shanghai Key Laboratory of Signaling and Disease Research, Collaborative Innovation Center for Brain Science, Frontier Science Center for Stem Cell Research, National Stem Cell Translational Resource Center, School of Life Sciences and Technology, Tongji University, Shanghai, China; Clinical and Translational Research Center of Shanghai First Maternity and Infant Hospital, Shanghai Key Laboratory of Signaling and Disease Research, Collaborative Innovation Center for Brain Science, Frontier Science Center for Stem Cell Research, National Stem Cell Translational Resource Center, School of Life Sciences and Technology, Tongji University, Shanghai, China; Clinical and Translational Research Center of Shanghai First Maternity and Infant Hospital, Shanghai Key Laboratory of Signaling and Disease Research, Collaborative Innovation Center for Brain Science, Frontier Science Center for Stem Cell Research, National Stem Cell Translational Resource Center, School of Life Sciences and Technology, Tongji University, Shanghai, China; Institute for Regenerative Medicine, Shanghai East Hospital, School of Life Sciences and Technology, Frontier Science Center for Stem Cell Research, Tongji University, Shanghai, China; Bio-X Institute, Shanghai Jiao Tong University, Shanghai, China; Clinical and Translational Research Center of Shanghai First Maternity and Infant Hospital, Shanghai Key Laboratory of Signaling and Disease Research, Collaborative Innovation Center for Brain Science, Frontier Science Center for Stem Cell Research, National Stem Cell Translational Resource Center, School of Life Sciences and Technology, Tongji University, Shanghai, China; Department of Biostatistics and Computational Biology, State Key Laboratory of Genetic Engineering, School of Life Sciences, Fudan University, Shanghai, China; Clinical and Translational Research Center of Shanghai First Maternity and Infant Hospital, Shanghai Key Laboratory of Signaling and Disease Research, Collaborative Innovation Center for Brain Science, Frontier Science Center for Stem Cell Research, National Stem Cell Translational Resource Center, School of Life Sciences and Technology, Tongji University, Shanghai, China; Clinical and Translational Research Center of Shanghai First Maternity and Infant Hospital, Shanghai Key Laboratory of Signaling and Disease Research, Collaborative Innovation Center for Brain Science, Frontier Science Center for Stem Cell Research, National Stem Cell Translational Resource Center, School of Life Sciences and Technology, Tongji University, Shanghai, China; Clinical and Translational Research Center of Shanghai First Maternity and Infant Hospital, Shanghai Key Laboratory of Signaling and Disease Research, Collaborative Innovation Center for Brain Science, Frontier Science Center for Stem Cell Research, National Stem Cell Translational Resource Center, School of Life Sciences and Technology, Tongji University, Shanghai, China; Clinical and Translational Research Center of Shanghai First Maternity and Infant Hospital, Shanghai Key Laboratory of Signaling and Disease Research, Collaborative Innovation Center for Brain Science, Frontier Science Center for Stem Cell Research, National Stem Cell Translational Resource Center, School of Life Sciences and Technology, Tongji University, Shanghai, China; Clinical and Translational Research Center of Shanghai First Maternity and Infant Hospital, Shanghai Key Laboratory of Signaling and Disease Research, Collaborative Innovation Center for Brain Science, Frontier Science Center for Stem Cell Research, National Stem Cell Translational Resource Center, School of Life Sciences and Technology, Tongji University, Shanghai, China; Institute for Regenerative Medicine, Shanghai East Hospital, School of Life Sciences and Technology, Frontier Science Center for Stem Cell Research, Tongji University, Shanghai, China; Clinical and Translational Research Center of Shanghai First Maternity and Infant Hospital, Shanghai Key Laboratory of Signaling and Disease Research, Collaborative Innovation Center for Brain Science, Frontier Science Center for Stem Cell Research, National Stem Cell Translational Resource Center, School of Life Sciences and Technology, Tongji University, Shanghai, China

## Abstract

Long noncoding RNAs (lncRNAs) play a wide range of roles in the epigenetic regulation of crucial biological processes, but the functions of lncRNAs in cortical development are poorly understood. Using human embryonic stem cell (hESC)-based 2D neural differentiation approach and 3D cerebral organoid system, we identified that the lncRNA *PAUPAR*, which is adjacent to *PAX6*, plays essential roles in cortical differentiation by interacting with PAX6 to regulate the expression of a large number of neural genes. Mechanistic studies showed that *PAUPAR* confers PAX6 proper binding sites on the target neural genes by directly binding the genomic regions of these genes. Moreover, PAX6 recruits the histone methyltransferase NSD1 through its C-terminal PST enrichment domain, then regulate H3K36 methylation and the expression of target genes. Collectively, our data reveal that the *PAUPAR*/PAX6/NSD1 complex plays a critical role in the epigenetic regulation of hESC cortical differentiation and highlight the importance of *PAUPAR* as an intrinsic regulator of cortical differentiation.

## INTRODUCTION

The central nervous system (CNS) arises from the dorsal epiblast of the vertebrate gastrula and is highly complex in structure and function (1). During neural development, the most rostral primary vesicle in the neural tube, which is called the prosencephalon, later generates the telencephalon and diencephalon (2). The developing telencephalon comprises two major regions, the pallium and the subpallium. The cerebral cortex, which is the central region that controls complex cognitive behaviors (3), arises from the pallium, whereas ganglionic eminences arise from the subpallium (4). Unlike that of rodents, human beings’ cerebral cortex is with fabulous size and complex structures, and among which, the neocortex is thought to be the newest evolved brain region (5). Development of the cerebral cortex includes the expansion of neuroepithelial cells (NEs) and neural progenitors (NPs), as well as the generation of postmitotic neurons (6). Human ESCs (hESCs)-derived neural differentiation system, especially cerebral organoids differentiation, provides a useful tool for studying the regulatory mechanism underlying the human cortical development (7,8).

LncRNAs are expressed in multiple regions of the brain, including the cerebral cortex (9), have emerged as having regionally enriched expression profiles and can regulate gene expression through acting as signal molecules, scaffolds for protein complexes or decoys (10). LncRNA *Paupar*, which is adjacent to transcriptional factor *Pax6*, is highly expressed in mouse brain (11). Previous studies showed that *Paupar* interacts with Pax6 to maintain the self-renewal of mouse neuroblastoma Neuro-2a cells (11). *Paupar* was also found to function as a scaffold forming the *Paupar*/KAP1/PAX6 complex to regulate the proliferation and neuronal differentiation of Neuro-2a cells (12). Knockdown of *Paupar* or Kap1 at the SVZ of newborn mice using in utero electroporation can disrupt olfactory bulb neurogenesis (12), suggesting the regulatory role of *Paupar* in neural system. However, the roles of *PAUPAR* in telencephalon development remains elusive.

Several studies have shown that adjacent transcription factors and lncRNAs may interact closely and coordinate in diverse biological processes (13). *PAUPAR* is an antisense RNA upstream of the transcription factor *PAX6* that plays an important role in development of the nervous system. In mouse neural development, Pax6 is first detected in the developing forebrain at around E8.0, 1 day after NEs formation (14,15). Unlike mouse homolog, human PAX6 is expressed as early as in the developing telencephalon from the beginning of neural plate formation and functions as a major determinant of human neural development. Using a human pluripotent stem cell neural differentiation system, Zhang *et al.* found that *PAX6* was essential for the differentiation of epiblast cells into NEs, and knockdown of *PAX6* could decisively impair NE specialization and neurogenesis (16). However, it is unclear whether there is interaction between *PAUPAR* and PAX6, and the role of this interaction in regulating the differentiation of human dorsal telencephalon.

Formation of complexes with epigenetic enzymes serves as an important way for lncRNAs and transcription factors to coordinately regulate the epigenetic modification of chromatin and hence facilitate downstream genes expression (13,17). A chromatin signature called ‘K4-K36 domain’, which consists of the promoter region with H3K4me3 and the gene body region with H3K36me3, marks actively transcribed genes (18). A series of H3K36 methyltransferases, including NSD1, catalyze the formation of H3K36me2, and then to H3K36me3 under the catalysis of SETD2. NSD1 controls the H3K36 methylation level within and surrounding the gene body, knockdown NSD1 can decrease all three forms of methylated H3K36 and reduce the downstream genes expression (19). The role of NSD1 in the gene regulation of *PAUPAR* and PAX6 remains unknown.

Here, we show that the lncRNA *PAUPAR* is required for cortical differentiation and significantly affects cortical neural gene expression by functionally interacting with PAX6 and directing PAX6 to downstream target genomic regions. Further studies revealed that PAX6 could recruit the H3K36 methylation enzyme NSD1 to form a *PAUPAR*/PAX6/NSD1 complex that regulates the expression of target genes in hESC cortical differentiation.

## MATERIALS AND METHODS

### hESC culture

H9 hESCs (WiCell Institute, Madison, WI, USA, passages 25–55) were cultured on a feeder layer of irradiated mouse embryonic fibroblasts (MEFs) as described in a standard protocol (http://www.wicell.org). The hESC culture medium, consisting of Dulbecco's modified Eagle's medium (DMEM)/F12 (Gibco), 20% Knockout serum replacement (Gibco), 0.1 mM β-mercaptoethanol (Amresco), 1% NEAA (GIBCO), 0.5% l-glutaMAX (Gibco) and 4 ng/ml FGF-2 (Sino Biological).

### 2D hESC neural differentiation

Neural differentiation of hESCs was performed as described previously (16). hESCs were incubated with dispase (Gibco) at 37°C. Then, colonies were cultured in suspension as embryoid bodies (EBs) in hESC culture medium without FGF-2. On day 4, the hESC culture medium was replaced with neural differentiation medium (DMEM/F12 (Gibco), N2 supplement (Gibco), 1% NEAA (Gibco) and 2 μg/ml heparin (Sigma)). On day 7, the EBs were attached to plastic substrate and cultured with neural differentiation medium for 6–8 days, and the columnar neuroepithelia organized into rosette structures. The rosette were physically resuspended and cultured in neural differentiation medium. On day 28, neural spheres were attached to plastic substrate or coverslips treated with polyornithine (Sigma) and Matrigel (BD Biosciences) to promote neuron formation. For ventral telencephalon differentiation, we treated neuroepithelial cells with 1.5 M SAG (Selleck) and 200 ng/ml Shh (R&D Systems) at days 10–25 as previously described (20).

### Generation of cerebral organoids

Cerebral organoids were generated as previously described (8) with minor modifications. Briefly, H9 hESCs were maintained as a feeder-free system in mTeSR1 medium (Stem Cell Technologies) and cultured on Matrigel (Corning)-coated plates. EBs were prepared from a 9000 single-cell suspension and cultured in mTeSR1 medium with RevitaCell (Gibco) in a 96-well ultralow attachment plat. On day 3, the medium was replaced with mTeSR1 medium without RevitaCell. On day 5 or 6, depending on their morphology, EBs were transferred into neural induction medium (DMEM/F12 (Gibco), N2 supplement (Gibco), 1% GlutaMAX (Gibco), 1% NEAA (Sigma) and 2 μg/ml heparin (Sigma)). On day 11, EBs were embedded in Matrigel (Corning) droplets and cultured with improved differentiation medium A (1:1 DMEM/F12 and neurobasal medium (Gibco), 0.5% N2 supplement, 2% B27 without vitamin A (Gibco), a 0.25% insulin solution (Sigma), 1% GlutaMAX, 0.5% MEM-NEAA, 0.1% sodium bicarbonate (Sigma) and 1% penicillin-streptomycin (Sigma)) without shaking for 5 days, followed by culture with fresh improved differentiation medium A on an orbital shaker for another 5 days. Then, the EBs were cultured with improved differentiation medium A (1:1 DMEM/F12 and neurobasal medium, 0.5% N2 supplement, 2% B27 with vitamin A (Gibco), a 0.25% insulin solution, 1% GlutaMAX, 0.5% MEM-NEAA, 0.1% sodium bicarbonate, 0.007% ascorbic acid (Sigma) and 1% penicillin-streptomycin) on an orbital shaker. Media were changed once weekly.

### Immunofluorescence staining

Cells cultured on coverslips were fixed with 4% paraformaldehyde at 4°C for 15 min and washed three times with PBS. The cells were blocked in PBS/10% donkey serum/0.1% Triton X-100 for 1 h at room temperature. Then, the samples were incubated with primary antibody overnight at 4°C and secondary antibody/HO 33342 for 1 h at room temperature. Fluorescent images were captured by fluorescence microscopy (Nikon). Quantitative analysis was performed using ImageJ (https://imagej.nih.gov/ij/) software. The antibodies used in this study are listed in Supplemental Table S1.

### RNA immunoprecipitation (RIP)

RIP assay was performed as described previously (13). Cells were lysed with RIP buffer (10 mM HEPES (pH 7.0), 100 mM KCl, 5 mM MgCl_2_, 0.5% NP-40 and 1 mM dithiothreitol) for 30 min on ice. The proteins were immunoprecipitated using control IgG (CST) or anti-PAX6 (Abcam) antibody. Coimmunoprecipitated RNAs were extracted using RNAiso (Takara) and analyzed by QRT-PCR. The primer sequences used in this study are listed in Supplemental Table S2.

### *In vitro* triplex pulldown assay

An *in vitro* triplex pulldown assay was performed as previously described with modifications (21). PCR fragments (SOX1: Piece1 –416/+90, Piece2 +60/+470, Piece3 +431/+1023, Piece4 +1074/+1713, Piece5 +1721/+2289, Piece6 +2305/+2760 and GAPDH +3094/+3583 as a negative control) were treated with exonuclease I (Thermo) and incubated with biotin-labeled PAUPAR mutants in 10 mM Tris (pH 7.5), 20 mM KCl, 10 mM MgCl_2_ and 0.05% Tween 20 supplemented with RNaseOUT at room temperature for 20 min. Then, the samples were incubated with prewashed streptavidin magnetic C1 beads (invitrogen), followed by three washes with wash buffer I (10 mM Tris (pH 7.5), 150 mM KCl, 5 mM MgCl_2_, 0.5% NP-40 and RNaseOUT) and one was with wash buffer II (10 mM Tris (pH 7.5), 15 mM KCl and 5 mM MgCl_2_). The beads were treated with elution buffer (50 mM Tris (pH 8.0), 1% SDS and 10 mM EDTA) at 65°C for 5 min. After RNaseA and proteinase K treatment, the recovered DNA was analyzed by QRT-PCR and normalized to input DNA. The primer sequences used in this study are listed in Supplemental Table S2.

### Cytoplasmic and nuclear RNA fractionation

Nuclear and cytoplasmic RNA was isolated as described previously (13). Cells were collected, washed with ice-cold PBS and centrifuged at 1000 rpm for 5 min. Cell pellets were gently resuspended and incubated in 200 μl of lysis buffer A (10 mM Tris (pH 8.0), 140 mM NaCl, 1.5 mM MgCl_2_ and 0.5% NP-40) on ice for 5 min. Then, the samples were centrifuged at 1000 × g for 3 min at 4°C. The supernatant, which contained the cytoplasmic fraction, was transferred into a new tube to which 1 μl of RNAiso reagent (Takara) was added. Nuclear pellets underwent two additional washes with lysis buffer A and lysis buffer A containing 1% Tween-20 and 0.5% deoxycholic acid. Purified nuclear pellets were finally resuspended in 1 ml of RNAiso reagent.

### Statistical analysis

The data in this study were expressed as the mean±error (SEM) from three independent experiments (*n* = 3). To quantify the immunofluorescence staining of cells expressing type-specific markers, at least 9 sections from at least three independent experiments were collected, and at least 1000 cells were counted. Significance was set at <0.05 (see each figure legend for details). ANOVA and Student's *t-*test statistical analyses were performed using GraphPad software (https://www.graphpad.com).

## RESULTS

### *PAUPAR* specifically regulates hESC cortical differentiation initiated by its nearby transcription factor *PAX6*

**Figure 1.**
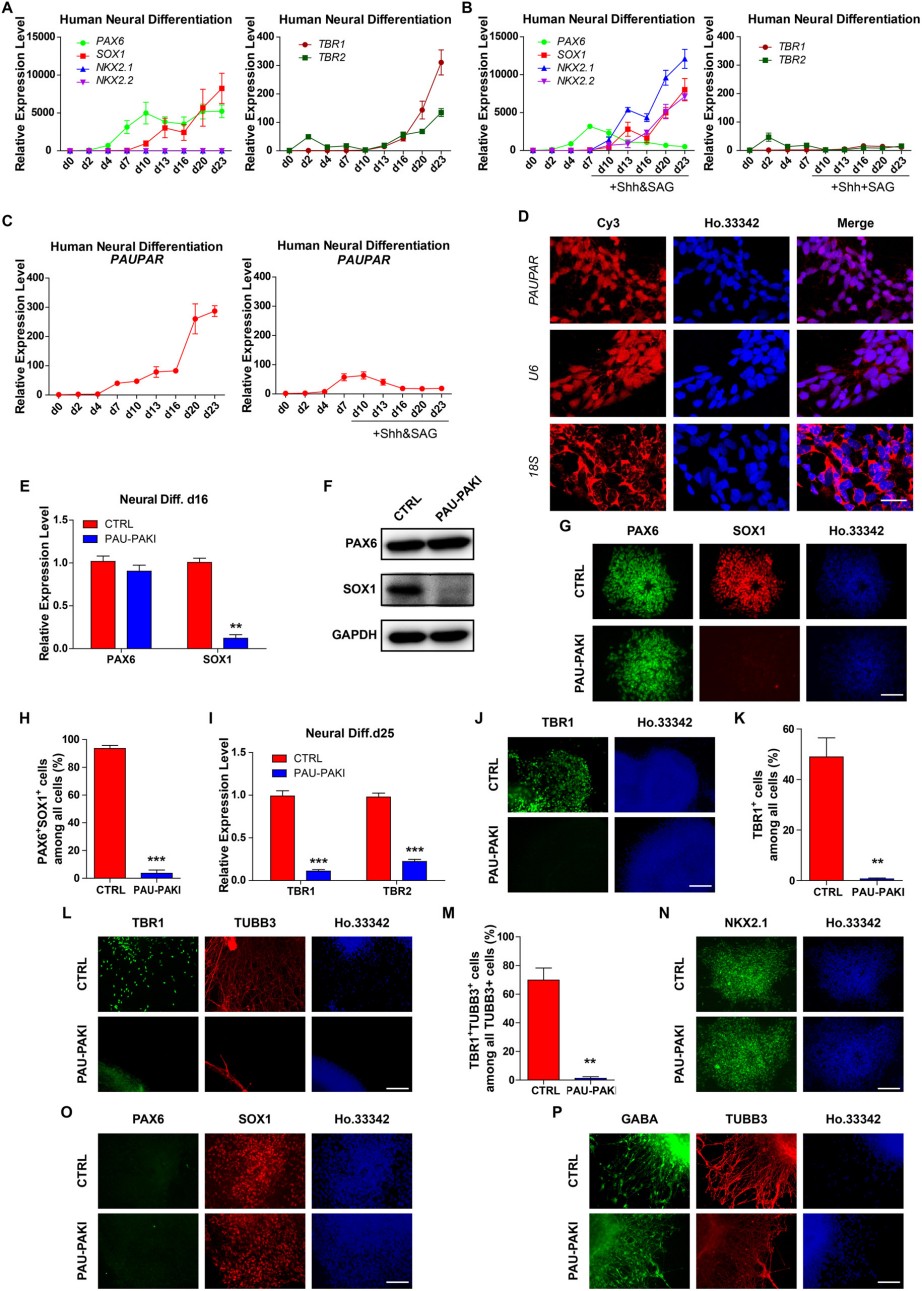
*PAUPAR* specifically regulates hESCs dorsal telencephalon differentiation but not ventral telencephalon differentiation. (A and B) Expression profiles of *PAX6*, *SOX1*, *NKX2.1*, *NKX2.2*, *TBR1* and *TBR2* during dorsal (**A**) and ventral (**B**) neural differentiation. (**C**) Expression profiles of *PAUPAR* during dorsal and ventral neural differentiation. (**D**) FISH with specific *PAUPAR* probes (LNC1100283, RiboBio) in day 16 NE. Human *U6* (LNC110101, RiboBio), positive control for nuclear RNA; human *18S* (LNC110102, RiboBio), positive control for cytoplasmic RNA. The nuclei were stained with Hoechst33342. Scale bar, 25 μm. (**E**, **F**) Expression of PAX6 and SOX1 mRNA level (E) and protein level (F) in control and PAU-PAKI cells on day 16 of dorsal neural differentiation. (**G**, **H**) Immunostaining assay of PAX6 (green) and SOX1 (red) in control and PAU-PAKI cells on day 16 of dorsal neural differentiation (G), and quantification of PAX6^+^SOX1^+^ cells (H). Scale bar, 100 μm. (**I**) Expression of TBR1 and TBR2 in control and PAU-PAKI cells on day 25 of dorsal neural differentiation. (**J**, **K**) Immunostaining assay of TBR1 (green) in control and PAU-PAKI cells on day 25 of dorsal neural differentiation (J), and quantification of TBR1^+^ cells (K). Scale bar, 100 μm. (**L**, **M**) Immunostaining assay of TBR1 (green) and TUBB3 (red) in control and PAU-PAKI cells on day 30 of dorsal neural differentiation (L), and quantification of TBR1^+^TUBB3^+^ cells among all TUBB3^+^ cells (M). Scale bar, 100 μm. (**N**) Immunostaining assay of NKX2.1 (green) in control and PAU-PAKI cells on day 25 of ventral neural differentiation. Scale bar, 100 μm. (**O**) Immunostaining assay of PAX6 (green) and SOX1 (red) in control and PAU-PAKI cells on day 16 of ventral neural differentiation. Scale bar, 100 μm. (**P**) Immunostaining assay of GABA (green) and TUBB3 (red) in control and PAU-PAKI cells on day 30 of ventral neural differentiation. Scale bar, 100 μm. Data are presented as mean ±SEM. *P< 0.05, **P< 0.01, ***P< 0.001 (*t*-test). See also Supplementary Figures S1 and S2.

Based on 2D neural differentiation protocol, hESCs can be efficiently differentiated into enriched PAX6-expressing NEs and further differentiated into dorsal forebrain and medial ganglionic eminence (MGE) NPs. Consistent with previous studies (22), the expression of early neural differentiation-related transcription factors *PAX6* increased from day (d) 2 and *SOX1* from d7 (Figure 1A). On the 23rd day of differentiation, the cells then expressed the dorsal forebrain-associated transcription factors *TBR1* and *TBR2*, but not the ventral NPs-related transcription factors, *NKX2.1* and *NKX2.2* (Figure 1A). Furthermore, by comparing the RNA expression profiles of hESC neural differentiation samples and the online RNA-Seq data of brain tissues of human embryos (23) (http://www.ebi.ac.uk/gxa/experiments/E-MTAB-4840), the gene expression patterns of the differentiation samples on d16 and d23 were similar to those of the forebrain and cortex tissues from 12-week-old human embryos (Supplementary Figure S1A), confirming that hESCs actually differentiated into NPs of the cerebral cortex during neural differentiation process. To differentiate hESC to the ventral forebrain MGE cells, ventralization factor sonic hedgehog (Shh) and SAG, a Smoothened agonist that can activate the Shh pathway were added from d10–25 neural differentiation (20). As expected, *PAX6* expression decreased with ventralized differentiation while *SOX1*, *NKX2.1* and *NKX2.2* expression increased, but no *TBR1* and *TBR2* expression (Figure 1B).

The lncRNA *PAUPAR* is an antisense RNA upstream of *PAX6* on chromosome 11. During hESC-based 2D neural differentiation, both *PAUPAR* and *PAX6* were observed to be upregulated in cortical differentiation but could not be detected in ventral differentiation (Figure 1C). Further, we found that *PAUPAR* was mainly located in the nucleus by fluorescent *in situ* hybridization (Figure 1D) and cytoplasmic/nuclear RNA fractionation analysis (Supplementary Figure S1B), and that *PAUPAR* bound with PAX6 in d16 cortical NEs by RIP analysis (Supplementary Figure S1C) and MS2bp-YFP RNA pull-down analysis (24) (Supplementary Figure S1D). To study the role of *PAUPAR* in hESC-based neural differentiation, a *PAUPAR* inhibition cell line (PAU-PAKI) was established through inserting three consecutive polyA transcription termination signals into the start site of *PAUPAR* by CRISPR/Cas9 (Supplementary Figure S1E-G) and performed the neural differentiation. We found that during the PAU-PAKI differentiation into NEs, the expression of the early neural transcription factors *OCT6*, *ZNF521* and *N-CAD* was not altered upon *PAUPAR* inhibition (Supplementary Figure S1H). Then in PAU-PAKI cortical differentiation, although not affecting the expression of *PAX6*, inhibition of *PAUPAR* significantly downregulated the expression of *SOX1*, and further significantly reduced the number of TBR1^+^ cells and TUBB3^+^/TBR1^+^ cortical neurons (Figure 1E–M). However, during differentiation into the ventral MGE cells, *PAUPAR* inhibition did not affect the expression of SOX1, and the cells differentiation into NKX2.1^+^ cells and GABA^+^ neurons (Figure 1N–P). These results indicate that inhibition of *PAUPAR* does not affect the differentiation of hESCs to NEs and further ventral forebrain differentiation, but significantly inhibits the cortical differentiation.

Since *PAUPAR* is adjacent to *PAX6*, a key regulator in neural differentiation. To check whether *PAUPAR* play similar roles as PAX6, the *PAX6* knockout cell line (PAX6KO) (22) was used to differentiate into dorsal forebrain and MGE respectively (Supplementary Figure S2A). Unlike *PAUPAR* inhibition, *PAX6* knockout significantly inhibited the expression of *OCT6*, *ZNF521* and *N-CAD* during the hESC differentiation into NEs (Supplementary Figure S1H). In cortical differentiation, similar with *PAUPAR* inhibition, *PAX6* knockout significantly decreased the expression of *SOX1* on d16 and the number of TBR1^+^ cells and TUBB3^+^/TBR1^+^ cortical neurons (Supplementary Figure S2B–H). On the contrary, in the ventral MGE differentiation, *PAX6* knockout showed different effect with *PAUPAR* inhibition. Although PAX6KO cells still could differentiate into NKX2.1^+^ cells, *PAX6* knockout significantly reduced the number of SOX1^+^ cells, and could not further differentiate the cells into GABA^+^ neurons (Supplementary Figure S2I–K). These results show that *PAX6* knockout in the initiation stage of neural differentiation can inhibit hESCs differentiation to NEs, further affects telencephalon differentiation of both the dorsal and ventral sides, while *PAUPAR* inhibition only affects the dorsal telencephalon differentiation.

Interestingly, we found that *PAX6* knockout downregulated *PAUPAR* expression level during cortical differentiation (Supplementary Figure S1G). Then to test how PAX6 regulated *PAUPAR* expression, the promoter region of *PAUPAR* (TSS −2000 to +1000) was cloned into the pGL3 vector and co-transfected with PAX6 into 293FT cells to perform the Dual-Luciferase reporter Assay. The result showed that PAX6 could bind the promoter region and activate the expression of *PAUPAR* (Supplementary Figure S2L). Further, chromatin immunoprecipitation sequencing (ChIP-seq) analysis of the hESC cortical differentiation sample at d16 showed that PAX6 could directly bind the transcription initiation region of *PAUPAR* and this binding was not affected by the inhibition of *PAUPAR* (Supplementary Figure S2M). In summary, these results indicate that *PAUPAR* is regulated by PAX6 and specifically regulates the hESC cortical differentiation.

### Deletion of *PAUPAR* impairs cortical differentiation in organoid culture

**Figure 2.**
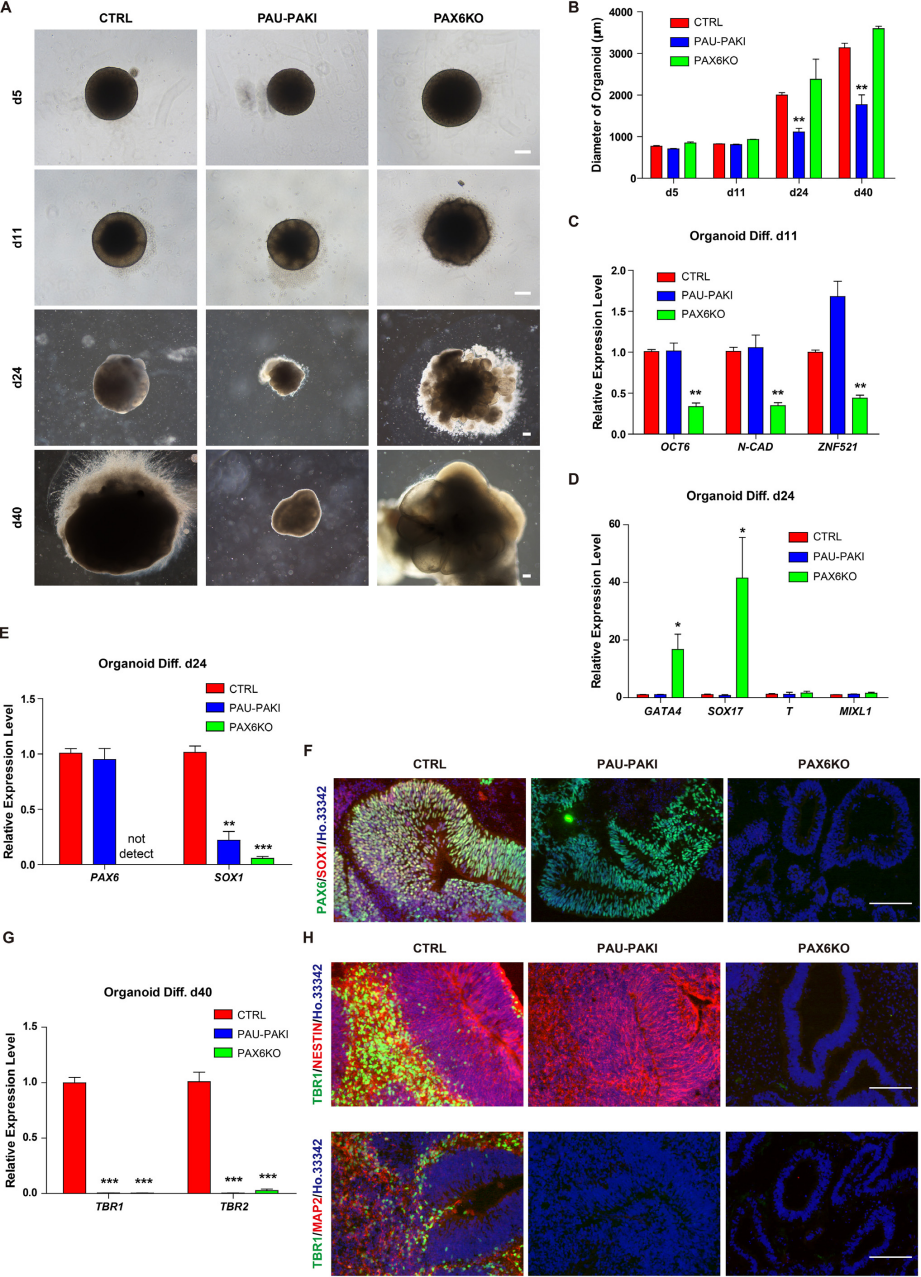
Inhibition of *PAUPAR* or knockout *PAX6* can impair dorsal telencephalon differentiation in organoids culture. (**A**) Example images of organoid differentiation for control, PAU-PAKI and PAX6KO cells on day 5, day 11, day 24 and day 40. Scale bar, 200 μm. (**B**) Diameter of organoids for control, PAU-PAKI and PAX6KO cells on day 5, day 11, day 24 and day 40. (**C**) Expression of *OCT6*, *N-CAD* and *ZNF521* in control, PAU-PAKI and PAX6KO organoids on day 11. (**D and E**) Expression of *GATA4*, *SOX17*, *T*, *MIXL1* (D) and *PAX6*, *SOX1* (E) in control, PAU-PAKI and PAX6KO organoids on day 24. (**F**) Immunostaining assay of PAX6 (green) and SOX1 (red) in control, PAU-PAKI and PAX6KO organoids on day 24. Scale bar, 100 μm. (**G**) Expression of TBR1 and TBR2 in in control, PAU-PAKI and PAX6KO organoids on day 40. (**H**) Immunostaining assay of TBR1 (green) and NESTIN (red) (up), TBR1 (green) and TUBB3 (red) (down) in control, PAU-PAKI and PAX6KO organoids on day 40. Scale bar, 100 μm. Data are presented as mean ± SEM. *P< 0.05, **P< 0.01, ***P< 0.001 (*t*-test).

The hESC-derived cerebral organoids provide an *in vivo*-like three-dimensional (3D) model that recapitulates many aspects of early developing human brains, especially the development course of cerebral cortex (8). To confirm our observations from hESC-based 2D neural differentiation in an in vivo-like system, we cultured human cerebral organoids from PAU-PAKI, PAX6KO as well as control H9 hESC lines. We found that at the initial stage of differentiation (d5), there was no difference in the embryoid body (EB) formation among the PAU-PAKI group, PAX6KO group and the control group (Figure 2A and B). However, on d11, the formation of NE-like cells could be observed in the outer side of spheres in the control group and the PAU-PAKI group, but not in the PAX6KO group (Figure 2A). Further investigation showed that, on d24, the expression of early neural genes including *OCT6*, *ZNF521* and *N-CAD* was downregulated in PAX6KO group, but not in PAU-PAKI group, compared to the control group (Figure 2C). These results confirmed the finding from hESC-based 2D neural differentiation that *PAX6* knockout could inhibit hESCs differentiation to NEs, while *PAUPAR* inhibition did not affect that. The more broad effect of PAX6KO than PAU-PAKI could be also proven by that endodermal genes *GATA4* and *SOX17* were upregulated in PAX6KO group but not PAU-PAKI group on d24 (Figure 2D). Further, we found that following the organoid culture, the diameter of organoids continuously increased, while in the PAU-PAKI organoids, the diameter of the organoids was significantly smaller than that of the control organoids (Figure 2B), suggesting that PAU-PAKI may impair the further differentiation of NEs to cortical NPs. Similar to 2D differentiation, *SOX1* expression was down regulated in PAU-PAKI group and PAX6KO group compared to control organoids (Figure 2E and F). On d40, *TBR1* and *TBR2* expression was downregulated in PAU-PAKI group and PAX6KO group (Figure 2G). Further immunofluorescence staining showed in the control group, a substantial number of NESTIN^+^ neural tube-like structures surrounded by TBR1^+^ cells and a large number of TBR1^+^/MAP2^+^ cortical neurons were detected. Interestingly, in PAU-PAKI organoids, although NESTIN^+^ neural tube-like structure still could be formed, there were very few MAP2^+^ neurons around the neural tube, and no TBR1^+^ cells were detected (Figure 2H). In the PAX6KO group, NESTIN^+^ neural tube-like structures and TBR1^+^/MAP2^+^ cells were absent (Figure 2H). Combined with our 2D differentiation data, these results further prove that *PAX6* is very important for the differentiation of neural tube epithelial cells, while *PAUPAR* specifically regulates the further differentiation of neural tube epithelial cells into cerebral cortex.

### *PAUPAR* and *PAX6* coordinate the expression of a large number of neural genes

**Figure 3.**
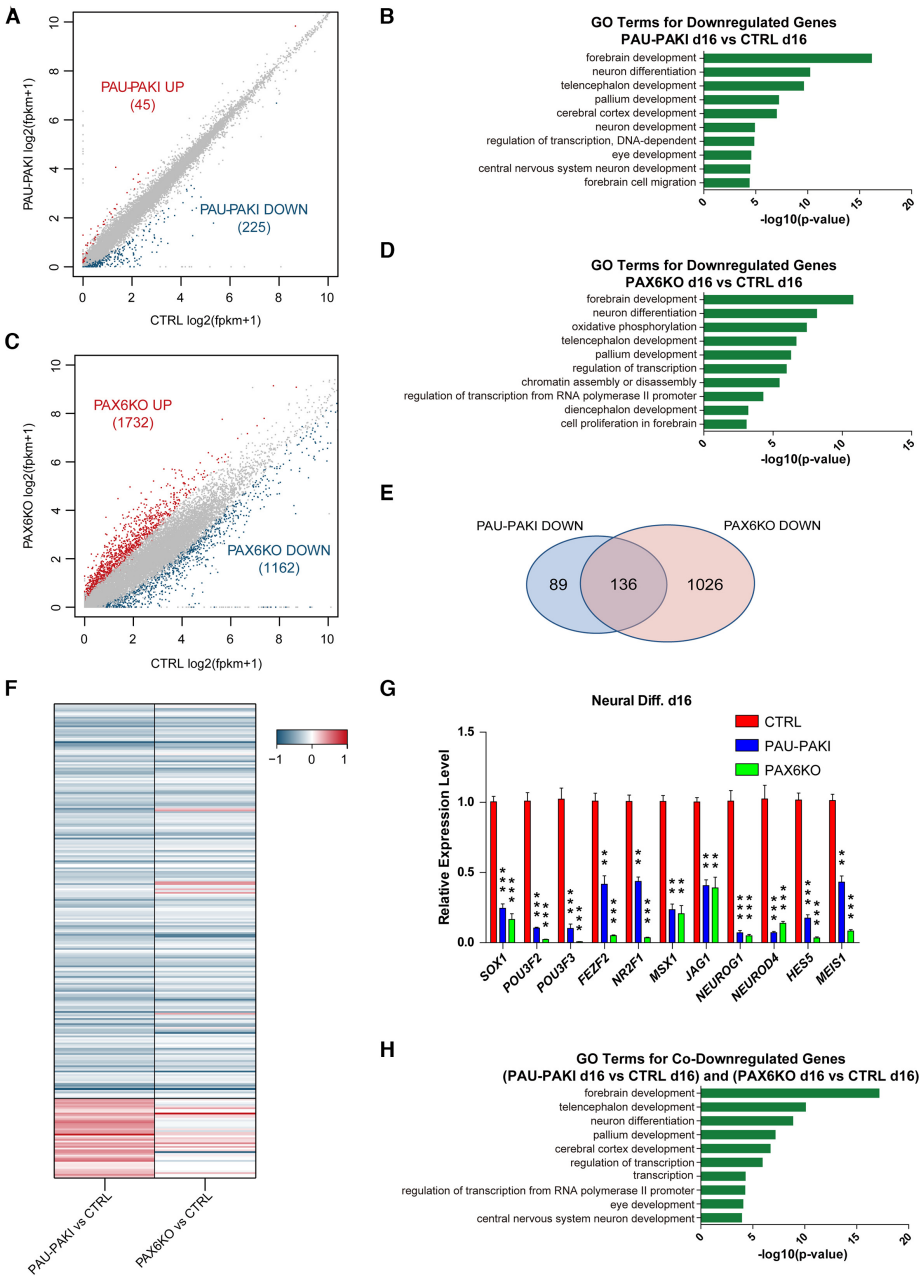
PAX6 and *PAUPAR* coordinately regulate the expression of a large number of neural genes. (**A**) RNA-seq scatterplot shows differential expression in control and PAU-PAKI cells on day 16 of dorsal neural differentiation. (**B**) Analysis of significant GO terms in genes that were downregulated by at least 2-fold in the PAU-PAKI cells compared with control cells. (**C**) RNA-seq scatterplot shows differential expression in control and PAX6KO cells on day 16 of dorsal neural differentiation. (**D**) Analysis of significant GO terms in genes that were downregulated by at least 2-fold in the PAX6KO cells compared with control cells. (**E**) The number of downregulated genes (at least2-fold) in PAU-PAKI cells and PAX6KO cells compared with control cells on day 16 of dorsal neural differentiation. (**F**) Heat map of significantly regulated genes in control, PAU-PAKI and PAX6KO cells on day 16 of dorsal neural differentiation. (**G**) Expression of representative downregulated genes related to neural differentiation (e.g. *SOX1*, *POU3F2*, *POU3F3*, *NR2F1* and *MSX1*) in control, PAU-PAKI and PAX6KO cells on day 16 of dorsal neural differentiation. (**H**) Analysis of significant GO terms in genes that were downregulated by at least 2-fold in both PAU-PAKI and PAX6KO cells compared with control cells. Data are presented as mean ± SEM. **P< 0.01, ***P< 0.001 (*t*-test). See also Supplementary Figure S3.

To study the mechanism by which *PAUPAR* regulates neural differentiation, RNA-seq was employed to quantitate gene expression in the control group, PAU-PAKI group and PAX6KO group on d16 dorsal neural differentiation. Compared with their expression in the control group, 45 genes were upregulated, while 225 genes were downregulated in the PAU-PAKI group (Figure 3A). Gene ontology (GO) analysis showed that the downregulated genes were mainly associated with neural differentiation, forebrain development, neuron differentiation, telencephalon development and cerebral cortex development (Figure 3B). Compared with their expression in the control group, 1732 genes were upregulated, while 1162 genes were downregulated in the PAX6KO group (Figure 3C). GO analysis showed that the downregulated genes were mainly related to biological processes such as forebrain development, oxidative phosphorylation, telencephalon development, and cell proliferation in the forebrain (Figure 3D). Further analysis showed that only 16 genes were upregulated in both PAU-PAKI and PAX6KO group, while 136 out of 225 downregulated genes in the PAU-PAKI group, including *SOX1*, *POU3F2*, *POU3F3*, *FEZF2*, *NR2F1*, *MSX1*, *JAG1*, *NEUROG1*, *NEUROD4*, *HES5* and *MEIS1*, were also downregulated in the PAX6KO group (Figure 3E–G and Supplementary Figure S3A). GO analysis showed that these 136 genes function mainly in the biological processes of forebrain development, telencephalon development, neuron differentiation and cerebral cortex development (Figure 3H). These analyses indicated that *PAUPAR* and *PAX6* jointly regulate the expression of a series of downstream neural genes during the cortical differentiation of hESCs. To verify if *PAUPAR* and *PAX6* coordinately regulate neural genes expression, we employed TALEN to construct hESC lines with the doxcycline inducible pTREtight promoter (pt) drove overexpression of PAX6a (H9 ptPAX6a), *PAUPAR* (H9 ptPAU) and the combination of PAX6a and *PAUPAR* (H9 ptPAX6a & PAU) (25) (Supplementary Figure S3B). At the stem cell stage, adding doxcycline for 5 days in the culture medium of H9 ptPAX6a & PAU could induce the expression of genes that were found to be jointly regulated by PAX6 and *PAUPAR*, such as *SOX1* and *POU3F3*, but in H9 ptPAX6a or H9 ptPAU could not (Supplementary Figure S3B). Furthermore, we found that only coexpression of PAX6a and *PAUPAR* could induce the SOX1 protein expression by western blot analysis (Supplementary Figure S3C), and the appearance of SOX1^+^ cells by immunofluorescence staining (Supplementary Figure S3D-E). These results confirmed that *PAX6* and *PAUPAR* coordinately regulate the expression of important neural genes.

### *PAUPAR* confers PAX6 proper binding site on the target neural genes

**Figure 4.**
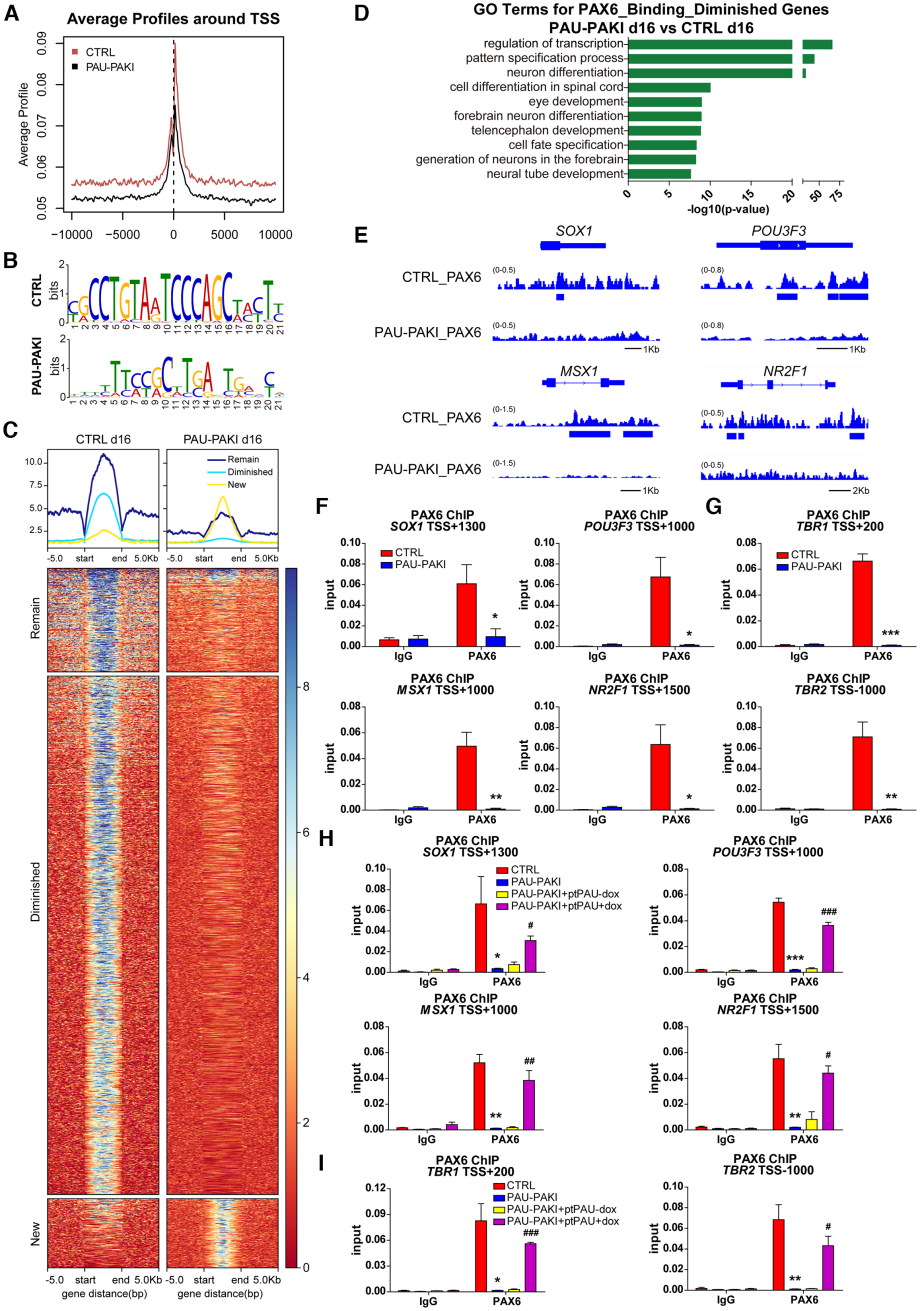
*PAUPAR* regulates PAX6 proper binding to the target neural genes. (**A**) Average profiles of PAX6 enrichment in genome-wide level of control and PAU-PAKI cells on day 16 of dorsal neural differentiation. (**B**) PAX6 binding motif in control and PAU-PAKI cells on day 16 of dorsal neural differentiation. (**C**) Genome-wide heatmaps of PAX6 enrichment of target genes in control and PAU-PAKI cells on day 16 of dorsal neural differentiation. (**D**) Analysis of significant GO terms in PAX6 binding diminished genes. (**E**) Genome browser screenshots of PAX6 ChIP-seq on *SOX1*, *POU3F3*, *MSX1* and *NR2F1* in control and PAU-PAKI cells on day 16 of dorsal neural differentiation. (**F**) Enrichment of PAX6 on *SOX1* (TSS +1300), *POU3F3* (TSS +1000), *MSX1* (TSS +1000) and *NR2F1* (TSS +1500) genomic region in control and PAU-PAKI cells on day 16 of dorsal neural differentiation. (**G**) Enrichment of PAX6 on *TBR1* (TSS +200) and *TBR2* (TSS –1000) genomic region in control and PAU-PAKI cells on day 25 of dorsal neural differentiation. (**H**, **I**) Enrichment of PAX6 on *SOX1* (TSS +1300), *POU3F3* (TSS +1000), *MSX1* (TSS +1000) and *NR2F1* (TSS +1500) genomic region after *PAUPAR* overexpression in the PAU-PAKI cells on day 16 (H), *TBR1* (TSS +200) and *TBR2* (TSS -1000) genomic region on day 25 (I) of dorsal neural differentiation. Data are presented as mean ± SEM. *P< 0.05, **P< 0.01, ***P< 0.001 versus CTRL group (F–I); ^#^*P*< 0.05, ^##^*P*< 0.01, ^###^*P*< 0.001 versus PAU-PAKI + ptPAU-dox group (H and I) (*t*-test). See also Supplementary Figure S4.

To further investigate the mechanism of *PAUPAR* and PAX6 coordinate regulation of gene expression, we detected the genomic binding sites of PAX6 in d16 of dorsal neural differentiation. ChIP-seq analysis showed that after the inhibition of *PAUPAR*, the binding of PAX6 to downstream genes was weakened (Figure 4A), and the binding motif of PAX6 changed (Figure 4B). After the inhibition of *PAUPAR*, the binding of PAX6 to 78.9% of genes was diminished, only 9.9% binding genes remained unchanged, while new binding on 11.2% of genes were generated (Figure 4C and Supplementary Figure S4A). The genes to which PAX6 binding was weakened mainly participate in the processes of neural differentiation and development (Figure 4D), these genes include *SOX1*, *POU3F3*, *MSX1* and *NR2F1* (Figure 4E and F). On d25 of neural differentiation, PAX6 was also found to bind the cortical genes *TBR1* and *TBR2*, but this binding activity of PAX6 was significantly weakened after the inhibition of *PAUPAR* (Figure 4G). These results suggest that *PAUPAR* is involved in regulating PAX6 binding to downstream neural gene regions.

For the *PAUPAR* rescue study, a doxcycline inducible *PAUPAR* overexpression cell line was established in PAU-PAKI cells (PAU-PAKI + ptPAU) (Supplementary Figure S4B). During dorsal neural differentiation, we found that overexpression of full-length *PAUPAR* from d8 to d25 could restore the expression of *SOX1*, *POU3F3*, *MSX1* and *NR2F1* on d16 (Supplementary Figure S4C–E), the expression of *TBR1* and *TBR2* on d25 (Supplementary Figure S4F–H), and the binding of PAX6 to these genes (Figure 4H and I). On d30 of differentiation, overexpression of full-length *PAUPAR* could also restore the proportion of TUBB3^+^/TBR1^+^ cortical neurons (Supplementary Figure S4I–J). These results indicate that the regulatory role of *PAUPAR* in cortical differentiation may be attribute to guiding PAX6 binding to downstream neural gene regions.

The catalytically inactive Cas9 (dCas9) protein can be used to target specific genome loci by using guide RNA that recognize target DNA sequences (26). To further detect the effect of guiding PAX6 binding to neural gene regions on the expression of these genes, a doxcycline inducible PAX6a/dCas9 fusion protein expression cell line was established in PAU-PAKI cells (PAU-PAKI + ptPAX6a/dCas9) and designed a guide RNA (gRNA) targeting the *SOX1* genome region (26). During dorsal neural differentiation, overexpression PAX6a/dCas9 along (d8–16) without gRNA could not active SOX1 expression, whereas collectively overexpression of gRNA and PAX6a/dCas9 (d8–16) could restore the expression of SOX1 even in the absence of *PAUPAR* (Supplementary Figure S4K–M), clearly proving that PAX6 does not bind the target neural gene region by itself and the guiding PAX6 to neural gene region is critical for these genes expression.

### *PAUPAR* directly binds to the genomic regions of target neural genes

**Figure 5.**
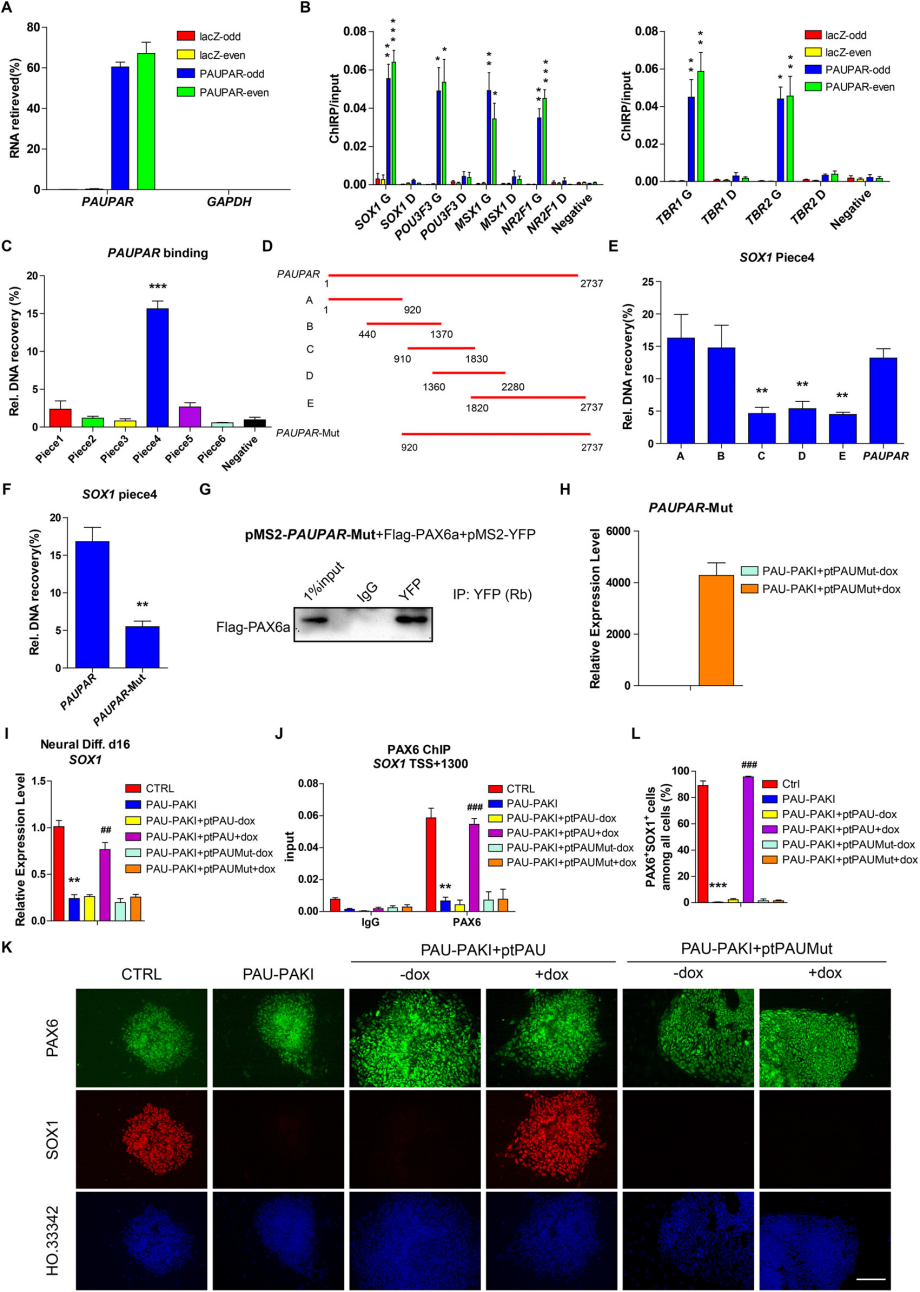
*PAUPAR* directly binds to genomic region of target neural genes. (**A**) Percent recovery of *PAUPAR* following ChIRP on day 16 of dorsal neural differentiation. *GAPDH* served as negative control. (**B**) ChIRP assay using probes targeting *PAUPAR* or *LacZ* mRNA followed by QRT-PCR on *SOX1* genome (G) (TSS +1300), *SOX1* distal region (D) (TSS -4000), *POU3F3* G (TSS +1000), *POU3F3* D (TSS -4000), *MSX1* G (TSS +1000), *MSX1* D (TSS –4000), *NR2F1* G (TSS +1500) and *NR2F1* D (TSS –4000) genomic region on day 16 (left); *TBR1* G (TSS +200), *TBR1* D (TSS –4000), *TBR2* G (TSS -1000) and *TBR2* D (TSS -4000) genomic region on day 25 (right) of dorsal neural differentiation. *GAPDH* (TSS +3500) served as negative control. (**C**) *In vitro* triplex pulldown assay showed the binding between *PAUPAR* and *SOX1* piece1–6 fragments (Piece1 -416/+90, Piece2 +60/+470, Piece3 +431/+1023, Piece4 +1074/+1713, Piece5 +1721/+2289, Piece6 +2305/+2760). *GAPDH* fragment (+3094/+3583) served as negative control. (**D**) Schematic representation of the deletion mutants of *PAUPAR*. (**E**) *In vitro* triplex pulldown assay showed the binding between *SOX1* piece4 fragment and full-length *PAUPAR* or *PAUPAR* deletion mutants (A, B, C, D, or E). (**F**) *In vitro* triplex pulldown assay showed the binding between *SOX1* piece4 fragment and full-length *PAUPAR* or *PAUPAR* 5′ region deletion mutant. (**G**) MS2bp-YFP RNA pull-down analysis showed the binding between Flag-PAX6a and *PAUPAR* 5′ region deletion mutant in 293FT extracts. (**H**) QRT-PCR analysis showed the overexpression of *PAUPAR* deletion mutant in the PAU-PAKI cells. (**I**) Expression of *SOX1* after *PAUPAR* or *PAUPAR* deletion mutant overexpression in the PAU-PAKI cells on day 16 of dorsal neural differentiation. (**J**) Enrichment of PAX6 on *SOX1* (TSS +1300) genomic region after *PAUPAR* or *PAUPAR* deletion mutant overexpression in the PAU-PAKI cells on day 16 of dorsal neural differentiation. (**K**, **L**) Immunostaining assay of PAX6 (green) and SOX1 (red) after *PAUPAR* or *PAUPAR* deletion mutant overexpression in the PAU-PAKI cells on day 16 of dorsal neural differentiation (K), and quantification of PAX6^+^SOX1^+^ cells (L). Scale bar, 100 μm. Data are presented as mean ± SEM. *P< 0.05, **P< 0.01, ***P< 0.001 versus *GAPDH* group (B), or versus negative group (C), or versus *PAUPAR* group (E and F), or versus CTRL group (I, J and L); ^##^*P*< 0.01, ^###^*P*< 0.001 versus PAU-PAKI + ptPAU-dox group (I, J and L) (*t*-test/ANOVA). See also Supplementary Figure S5.

Chromatin isolation by RNA purification (ChIRP) is a method to discover the RNA-bound DNA, then we performed ChIRP to test whether *PAUPAR* acted as a guide through binding to genome locus of neural genes (27). A set of probes target *PAUPAR* was designed and grouped into ‘‘even’’ and ‘‘odd’’ sets based on their positions along the *PAUPAR* RNA. A set of probes that targeted the lacZ mRNA was used as a negative control. Both ‘‘even’’ and ‘‘odd’’ *PAUPAR* probes could retrieve *PAUPAR* RNA but not *GAPDH* RNA and the lacZ probes could not retrieve *PAUPAR* RNA, demonstrating the specificity of *PAUPAR* probes (Figure 5A). The ChIRP results showed that *PAUPAR* was enriched on the genomic loci in the genome region of the PAX6 target genes *SOX1*, *POU3F3*, *MSX1* and *NR2F1* on d16, *TBR1* and *TBR2* on d25, but not in the distal region of these genes (Figure 5B). To examine if *PAUPAR* directly binds with target gene DNA, we incubated a DNA fragment comprising different regions of *SOX1* genomic DNA (Supplementary Figure S5A) with biotinylated *PAUPAR* RNA to perform an *in vitro* triplex pulldown assay (21), the results of pulldown assay showed that *PAUPAR* RNA could directly and specifically bind to the Piece4 of *SOX1* genomic DNA (Figure 5C). Then, we constructed a series of *PAUPAR* deletion mutants to detect which region of *PAUPAR* directly binds to the Piece4 fragment of *SOX1* genomic DNA, and found fragment A (nt 1–920) and fragment B (nt 440–1370) of *PAUPAR* could directly bind to *SOX1* genomic DNA (Figure 5D-E). Next, we constructed a *PAUPAR* mutant in which the 5′ region was deleted (*PAUPAR*-Mut, lacking nt 1–920), and found that the *PAUPAR*-Mut lost most of the *SOX1* genomic DNA binding capacity in *in vitro* triplex pulldown assay, but kept the capacity of binding PAX6 in MS2bp-YFP RNA pull-down analysis (Figure 5F and G). Through a functional assay, we found that overexpression of the mutant *PAUPAR* (PAU-PAKI+ptPAUMut) from d8 to d16 in PAU-PAKI cells did not restore SOX1 expression or PAX6 binding to *SOX1* gene region (Figure 5H–L), indicating that the genome binding of *PAUPAR* is vital to its function in regulating gene expression and that *PAUPAR* mainly relies on its 5′ region to bind to *SOX1* genomic DNA.

PAX6 contains three functional domains: the PD domain, HD domain and C-terminal PST enrichment domain (28). To investigate which domain is essential for PAX6 binding with *PAUPAR*, we constructed individual PAX6 deletion mutants lacking the PD domain (ΔPD, with amino acids 2–128 deleted), HD domain (ΔHD, with amino acids 210–272 deleted), or C-terminus (ΔC, with amino acids 303–422 deleted) and detected the binding between these mutants and *PAUPAR* by RNA pulldown. Deletion of the PD domain caused PAX6 to lose its ability to bind *PAUPAR*, but deletion of the HD domain or C-terminus did not affect this process (Supplementary Figure S5B). Further, full-length *PAX6* (PAX6KO+ptPAX6a) and a *PAX6* PD domain deletion mutant (PAX6KO + ptΔPD) was individually overexpressed in PAX6KO cells (Supplementary Figure S5C). Inducible overexpression of full-length *PAX6* (d8–25) could restore the mRNA levels of *SOX1, POU3F3, NR2F1* and *MSX1* on d16 as shown by QRT-PCR (Supplementary Figure S5D), and restore the proportion of SOX1^+^ cells as shown by immunofluorescence staining (Supplementary Figure S5E–F). Upon further differentiation to d25, the overexpression of *PAX6* restored the expression levels of *TBR1* and *TBR2* (Supplementary Figure S5G) as well as the number of TBR1^+^ cells (Supplementary Figure S5H–I). In contrast, inducible overexpression of the PD deletion mutant (d8–25) did not restore these neural differentiation defects (Supplementary Figure S5C-I). These results showed that the PD domain is essential for PAX6 binding with *PAUPAR* and the binding between *PAUPAR* and PAX6 is the main reason for their location on downstream gene regions and ability to regulate expression of these genes.

### *PAUPAR* and PAX6 regulate H3K36 methylation of the coding regions of downstream neural genes

**Figure 6.**
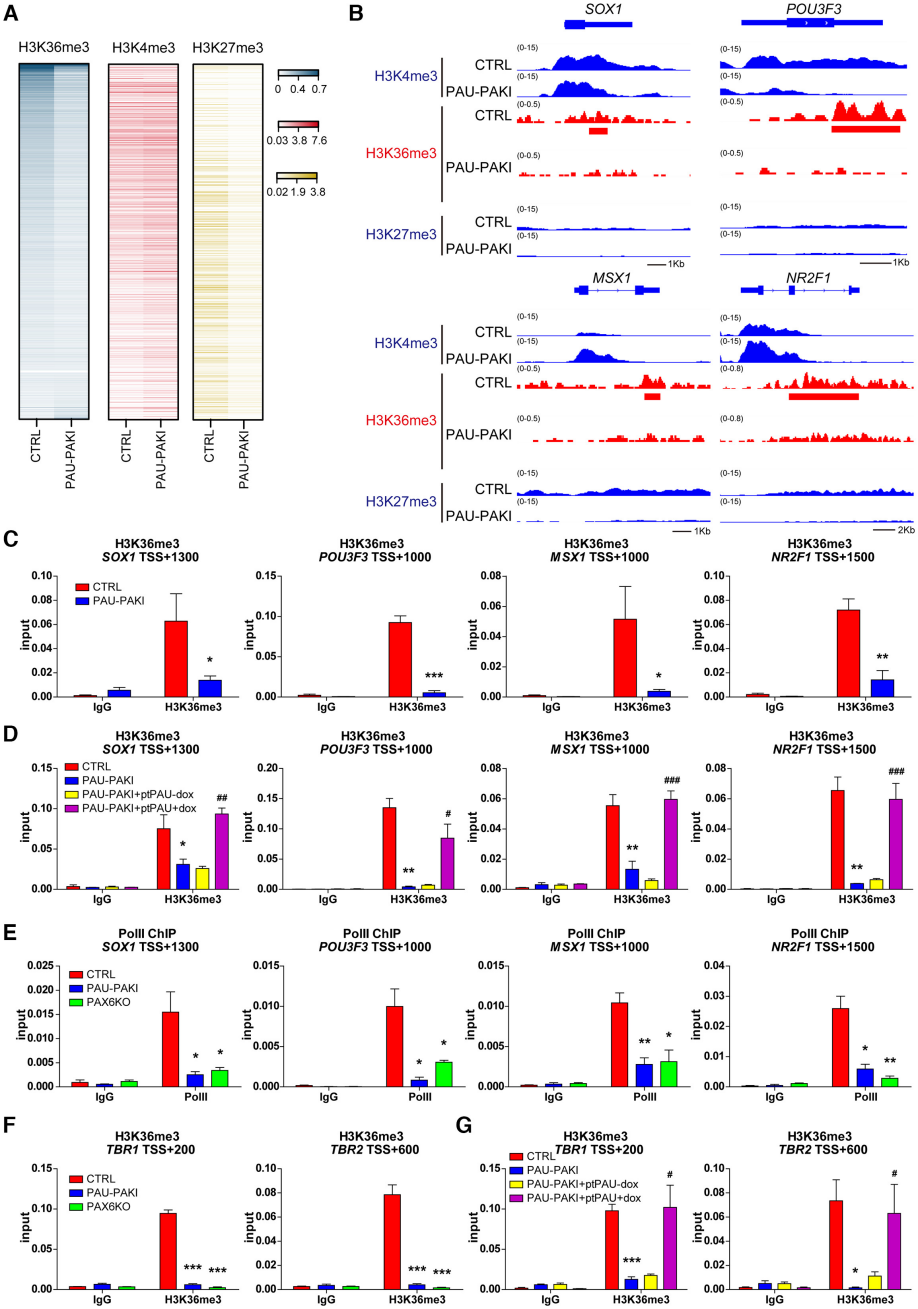
*PAUPAR* and PAX6 regulate H3K36 methylation in the coding region of downstream neural genes. (**A**) Enrichment levels of H3K36me3, H3K4me3 and H3K27me3 at PAX6 binding diminished genes on day 16 of dorsal neural differentiation. (**B**) Genome browser screenshots of H3K4me3, H3K36me3 and H3K27me3 ChIP-seq on *SOX1*, *POU3F3*, *MSX1* and *NR2F1* in control and PAU-PAKI cells on day 16 of dorsal neural differentiation. (**C**, **D**) Enrichment of H3K36me3 on *SOX1* (TSS +1300), *POU3F3* (TSS +1000), *MSX1* (TSS +1000) and *NR2F1* (TSS +1500) genomic region in control and PAU-PAKI cells (C), after *PAUPAR* overexpression in the PAU-PAKI cells (D) on day 16 of dorsal neural differentiation. (**E**) Enrichment of RNA-PolII on *SOX1* (TSS +1300), *POU3F3* (TSS +1000), *MSX1* (TSS +1000) and *NR2F1* (TSS +1500) genomic region in control, PAU-PAKI and PAX6KO cells on day 16 of dorsal neural differentiation. (**F and G**) Enrichment of H3K36me3 on *TBR1* (TSS +200) and *TBR2* (TSS +600) genomic region in control, PAU-PAKI and PAX6KO cells (F), after *PAUPAR* overexpression in the PAU-PAKI cells on day 25 of dorsal neural differentiation (G). Data are presented as mean ± SEM. *P< 0.05, **P< 0.01, ***P< 0.001 versus CTRL group (C–G); ^#^*P*< 0.05, ^##^*P*< 0.01, ^###^*P*< 0.001 versus PAU-PAKI + ptPAU-dox group (D and G) (*t*-test/ANOVA). See also Supplementary Figure S6.

Histone modifications, especially histone methylation, such as the decreased H3K4me3 and H3K36me3 modification of transcriptionally active sites and elevated H3K27me3 modification in the transcriptional inhibition region, are important causes of the downregulation of gene expression (29). ChIP-seq analysis on d16 cortical differentiation showed the gene regions that PAX6 binding diminished in the absence of *PAUPAR* were no significant difference in the H3K4me3 modification or not increased in H3K27me3 modification in *PAUPAR* inhibition group compared to that of the control group, but the level of H3K36me3 modification in these regions was significantly decreased (Figure 6A). For specific gene analysis, H3K27me3 modification levels of a series of specific neural genes jointly regulated by *PAUPAR* and PAX6, such as *SOX1*, *POU3F3*, *MSX1* and *NR2F1*, were low in the control and *PAUPAR* inhibition groups, while H3K4me3 modification levels were not obviously decreased (Figure 6B and Supplementary Figure S6A-B). And as expected, H3K36me3 modification levels in these neural genes were significantly decreased after the inhibition of *PAUPAR* (Figure 6B-C). Furthermore, overexpression of full-length *PAUPAR* (PAU-PAKI + ptPAU) from d8 to d25 of dorsal neural differentiation could restore the H3K36me3 modification of these neural genes (Figure 6D). Similarly, in PAX6KO cells, the H3K4me3 and H3K27me3 modification of *SOX1*, *POU3F3*, *MSX1* and *NR2F1* were also unchanged (Supplementary Figure S6C-D), whereas the H3K36me3 modification was significantly reduced (Supplementary Figure S6E). These results indicated that *PAUPAR* and PAX6 coregulate downstream neural gene expression by regulating H3K36me3 modification but not H3K27me3 or H3K4me3 modification. Consistent with the role of H3K36me3 as a histone modification signal associated with elongation by RNA polymerase II (30), the binding of RNA polymerase II to the gene bodies of these downstream neural genes was found to be reduced (Figure 6E), indicating that the transcriptional elongation of these genes was impaired after inhibition of *PAUPAR* or *PAX6* knockout. Upon further differentiation to d25, we found that the H3K36me3 modification level of *TBR1* and *TBR2* was also decreased after the loss of *PAUPAR* or *PAX6* (Figure 6F). Additionally, overexpression of full-length *PAUPAR* in PAU-PAKI cells could restore the H3K36me3 modification of *TBR1* and *TBR2* (Figure 6G). These results suggest that *PAUPAR*-mediated PAX6 binding to target neural genes is extremely important for H3K36me3 modification of these gene regions.

### *PAUPAR* and PAX6 specifically assemble with NSD1 to form a regulatory complex that governs local gene expression

**Figure 7.**
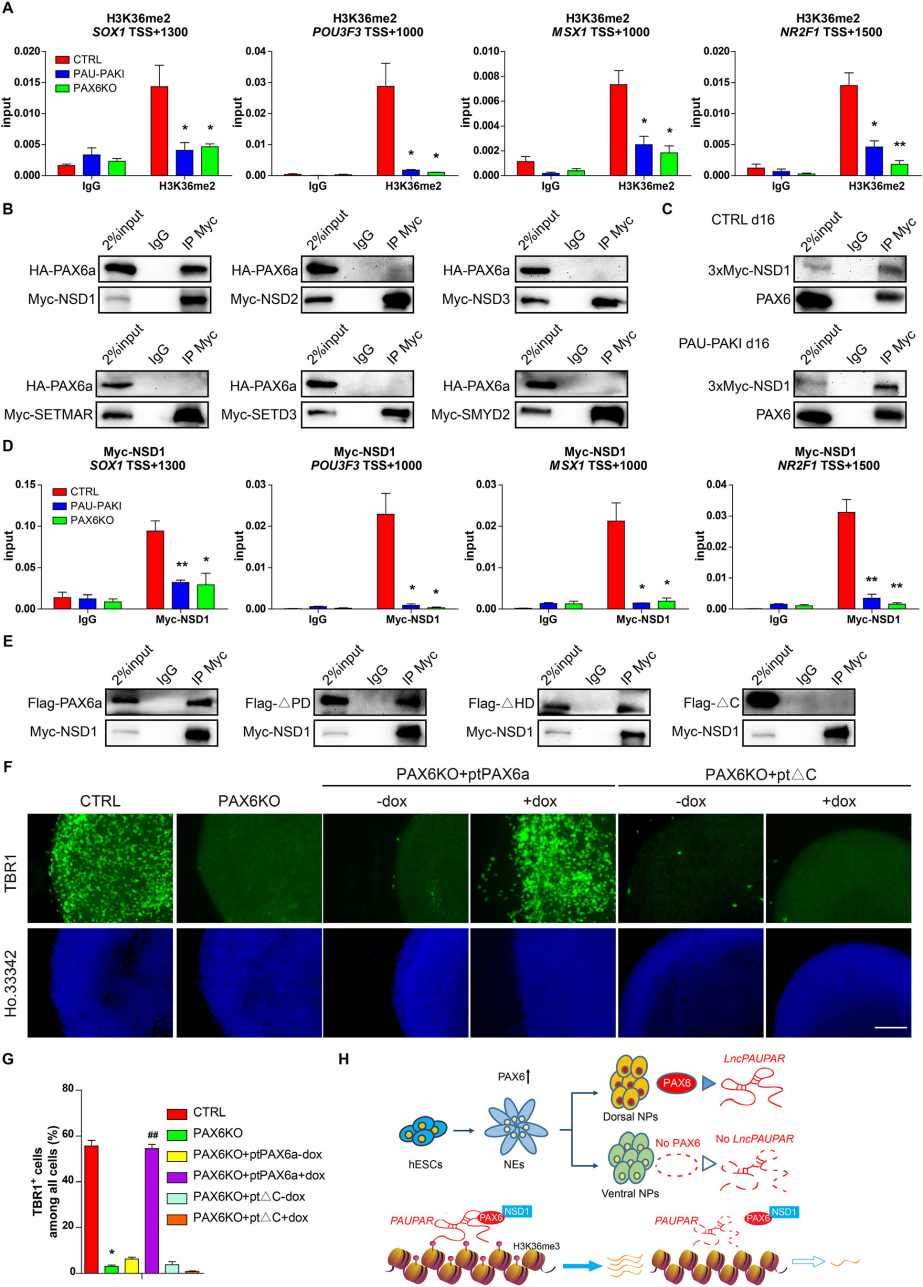
PAX6 interacts with NSD1 to regulate the H3K36me3 modification level and expression of the target neural genes. (**A**) Enrichment of H3K36me2 on *SOX1* (TSS +1300), *POU3F3* (TSS +1000), *MSX1* (TSS +1000) and *NR2F1* (TSS +1500) genomic region in control, PAU-PAKI and PAX6KO cells on day 16 of dorsal neural differentiation. (**B**) Co-IP analysis showing that HA-PAX6a interacts with myc-NSD1 in 293FT extracts, but not NSD2, NSD3, SETMAR, SETD3 or SMYD2. (**C**) Co-IP analysis showing that PAX6 interacts with myc-NSD1 in control and PAU-PAKI cells on day 16 of dorsal neural differentiation. (**D**) Enrichment of myc-NSD1 on *SOX1* (TSS +1300), *POU3F3* (TSS +1000), *MSX1* (TSS +1000) and *NR2F1* (TSS +1500) genomic region in control, PAU-PAKI and PAX6KO cells on day 16 of dorsal neural differentiation. (**E**) Co-IP analysis showing that myc-NSD1 interacts with Flag-PAX6a, Flag-ΔPD and Flag-ΔHD mutant in 293FT extracts, but not Flag-ΔC mutant. (**F**, **G**) Immunostaining assay of TBR1 (green) after *PAX6a* or ΔC mutant overexpression in the PAX6KO cells on day 25 of dorsal neural differentiation (F), and quantification of TBR1^+^ cells (G). Scale bar, 100 μm. (**H**) Summary of the *PAUPAR*/PAX6/NSD1-mediated transcriptional regulatory model. Data are presented as mean ± SEM. *P< 0.05, **P< 0.01 versus CTRL group (A, D and G); ^##^*P*< 0.01 versus PAX6KO + ptPAX6a-dox group (G) (*t*-test/ANOVA). See also Supplementary Figure S7.

Trimethylation of the lysine at position 36 of histone H3 is catalyzed in two steps. In the first step, the unmethylated lysine 36 is catalyzed to undergo monomethylation and then dimethylation. In this process, NSD1, NSD2, NSD3, SETD3, SETMAR and SMYD2 carry out catalytic activity. In the second step, SETD2 catalyzes the conversion of H3K36me2 to H3K36me3 (31). The H3K36me2 modification levels of *SOX1*, *POU3F3*, *MSX1* and *NR2F1* were significantly decreased after *PAUPAR* inhibition or *PAX6* knockout (Figure 7A), indicating that *PAUPAR* and PAX6 may participate in regulating H3K36 methylation in the first stage.

Coimmunoprecipitation (Co-IP) experiments in HEK293FT cells showed that PAX6 could bind NSD1, but not NSD2, NSD3, SETD3, SETMAR or SMYD2 (Figure 7B). Furthermore, due to lacking proper NSD1 antibody to perform the endogenous Co-IP and ChIP, three consecutive myc tags (3 × myc) were inserted to the C-terminal of NSD1 in one allele to pulldown endogenous NSD1 protein by anti-myc antibody (Supplementary Figure S7A), and Co-IP data showed binding between PAX6 and NSD1 was also detected in d16 of hESC cortical differentiation (Figure 7C). Further we found that although the loss of *PAUPAR* did not affect the binding between PAX6 and NSD1 (Figure 7C), the binding of NSD1 to *SOX1*, *POU3F3*, *MSX1* and *NR2F1* gene region was reduced by the inhibition of *PAUPAR* or *PAX6* knockout (Figure 7D). Upon further differentiation to d25, the binding of NSD1 to *TBR1* and *TBR2* gene region was also decreased after the inhibition of *PAUPAR* or *PAX6* knockout (Supplementary Figure S7B). To prove the importance of PAX6 and NSD1 binding, we further explored the domain responsible for PAX6 binding to NSD1. We found that the absence of the PD and HD domains did not affect the binding of PAX6 and NSD1, but loss of the C-terminus rendered PAX6 unable to bind NSD1 (Figure 7E), showing that the C-terminus of PAX6 is responsible for its binding of NSD1. Further, we found that overexpression of the *PAX6* C-terminus deletion mutant (PAX6KO + ptΔC) from d8 to d25 in the PAX6KO cell line (Supplementary Figure S7C) failed to restore the expression of *SOX1*, *POU3F3*, *MSX1*, or *NR2F1* on d16(Supplementary Figure S7D), the proportion of SOX1^+^ cells on d16 (Supplementary Figure S7E and F), the expression of *TBR1* and *TBR2* on d25 (Supplementary Figure S7G), or the generation of TBR1^+^ cells on d25 (Figure 7F and G), while overexpression of full-length *PAX6* from d8 to d25 successfully restored these neural differentiation defects (Figure 7F and G and Supplementary Figure S7C–G). Similarly, overexpression of the *PAX6* C-terminal deletion mutant did not restore the H3K36me3 modification at these gene regions, while overexpression of full-length *PAX6* could significantly restore the modification (Supplementary Figure S7H and I). These results indicate that the location of NSD1 in the PAX6/*PAUPAR* target neural gene regions is due to its binding to the C-terminus of PAX6, and interaction between PAX6 and NSD1 plays important roles in maintaining H3K36me3 modification at neural gene regions and hESC cortical differentiation.

## DISCUSSION

LncRNA *PAUPAR* is adjacent to transcription factor PAX6, whereas its expression pattern and potential function in human neural differentiation remains unrevealed. The hESC-derived brain organoids include the cortical progenitors with *in vivo*-like morphology and gene expression pattern, provide a promising platform to study the human cortical development (32,33). In the present study, our findings showed that *PAUPAR* expressed at the early stage of neural differentiation when the NEs formed, then specifically up-regulated in cortical differentiation. Inhibition of *PAUPAR* could lead to defects of cortical differentiation in cerebral organoid system. Previously, PAX6 is identified as the key regulator in cortical differentiation. Pax6 null mice died after birth with severely malformed cerebral cortex (34,35) and patients with PAX6 heterozygote mutations had greater decline in thickness of the frontoparietal cortex (36). In our study, we found that inhibition of *PAUPAR* did not affect the expression of PAX6, but impaired cortical differentiation, which means PAX6 alone was not sufficient for cortical differentiation. Further, our data showed that *PAUPAR* interacted with PAX6 and this interaction is critical for PAX6-mediated cortical differentiation, and in the stem cell stage, only overexpression of *PAUPAR* and PAX6 together could successfully initiate the expression of neural genes under the hESC culture condition. We also found the *PAUPAR* and PAX6 co-regulated genes were mostly enriched in those involved in telencephalon and cerebral cortex development, whereas in Neuro-2a cells, the genes controlled by both *Paupar* and Pax6 are enriched for regulators of synaptic functions (11), which indicated *PAUPAR* and PAX6 regulate diverse biological processes in hESC cortical differentiation and neuroblastoma cells. Collectively, we believe that our present findings firstly revealed the indispensable roles of *PAUPAR* as well as the interaction of *PAUPAR* and PAX6 in cortical differentiation.

LncRNAs play their regulatory roles through various mechanisms. Previous studies showed that, *Paupar* could regulate the alternative splicing of Pax6 5a, which was significantly enriched in α cells, in mouse pancreatic islet α cells (37). And *Paupar* could regulate the self-renewal of mouse Neuro-2a cells through acting as a scaffold to link PAX6 and KAP1 (12). Our findings firstly showed *PAUPAR* regulated the binding of PAX6 to downstream cortical related genes. ChIRP and *in vitro* triplex pulldown assays showed that *PAUPAR* directly bound downstream target gene genomic DNA, the mutated *PAUPAR* without the 5′ region failed to bring PAX6 to the genomic region of SOX1. By binding motif analysis of PAX6, we found that after *PAUPAR* was inhibited, the binding motif of PAX6 was similar to that reported by Jonathan et al., where the binding motif of PAX6 was identified by detecting the interaction between purified PAX6 protein and synthetic oligo-DNAs *in vitro* (38). Our study uncovered the novel function of *PAUPAR* that acting as a ‘guide’ to specifically mediate the binding of PAX6 to downstream gene regions during the cortical differentiation of hESC. Further study showed that PAX6 binds *PAUPAR* by its N-terminal PD domain, a DNA-binding region of PAX6 (39), means the special binding between PAX6 and its target genome DNA rely on the mediator effect of *PAUPAR* rather than the DNA binding ability of PD domain. This finding revealed a new molecular regulatory function of PD domain and also suggested that the binding specificity of transcription factor may be determined by the combination of transcription factor and its combined lncRNA.

In human neural development, PAX6 is one of the earliest regulators, which is expressed at the stage of NEs formation and essential for the epiblast cells differentiated into NEs (16). Our data showed that knocking out PAX6 at the beginning of neural differentiation, hESC could not enter neural Lineage. Instead, endoderm genes, such as SOX17 and GATA4 are upregulated in later differentiation. Unlike PAX6-deletion, inhibition of *PAUPAR* does not affect the early neural commitment. Human ESCs with *PAUPAR*-inhibition can differentiated into NESTIN-positive neural tube epithelial cells, indicating the role of PAX6 in cell fate commitment is independent of *PAUPAR*. Then in the process of cortical differentiation, unlike in mouse Neuro-2a cells where knocking down Pax6 does not affect *Paupar* expression (11), PAX6 directly bound to the promoter of *PAUPAR*, regulated its expression and interacted with *PAUPAR* regulating cortical genes expression. Those observation indicated the sequential roles of PAX6 and *PAUPAR* during human cortical development: as the key transcription factor, PAX6 is essential for neural commitment of hESC and inducing *PAUPAR*; *PAUPAR*, together with PAX6, manipulates further cortical differentiation.

We also identified a previously unrevealed PAX6 binding protein, the H3K36 methyltransferase NSD1. NSD1 is widely distributed in various tissues, including the brain and spinal cord, and that the absence of NSD1 leads to developmental defects in mice (40). In neural stem cell (NSC) differentiation, a decrease in the H3K36me3 modification of Gfap inhibited elongation of the Gfap gene by RNA polymerase II, thus reducing the expression of Gfap and promoting NSC differentiation to neurons (41). Our research showed that the *PAUPAR*/PAX6/NSD1 complex is located at the downstream neural genomic region, where it regulates the H3K36me3 modification and affects the elongation of target neural genes, therefore regulating their expression. Based on our knowledge, there is only one other *PAUPAR*/PAX6-contained complex was previously reported, that is the *Paupar*/PAX6/KAP1 complex in mouse Neuro-2a cells, where *Paupar* function as a scaffold and directly bound KAP1 and PAX6 (12). KAP1 is a critical chromatin regulator that involved in normal development and various pathological processes (42). Previous study showed that *Paupar*/PAX6/KAP1 complex regulates the H3K9me3 modification and silences downstream gene expression. That means the regulatory role of *PAUPAR* and PAX6 on downstream genes is differ based on recruiting different epigenetic enzymes in different biological processes.

In conclusion, our study revealed a new function of the lncRNA *PAUPAR* and a new complex composed of *PAUPAR*, PAX6 and NSD1 involved in the process of cortical differentiation (Figure 7H). These findings suggested that the formation of a specific lncRNA-transcription factor-epigenetic modifier complex allows them to participate in distinct gene transcription regulation and distinct differentiation and development process. This discovery will not only help us to better understand specific epigenetic regulation mediated by the *PAUPAR*/PAX6/NSD1 complex during neural differentiation, but also provides novel insights of regulatory mechanism in the development of human cerebral cortex.

## DATA AVAILABILITY

The accession number for the RNA-seq and ChIP-seq data reported in this paper is GSA database: CRA002482 (Shared URL: https://bigd.big.ac.cn/gsa/s/t1GkivqZ).

## Supplementary Material

gkab030_Supplemental_File
