# Supplementary material for: PAUPAR and PAX6 sequentially regulate human embryonic stem cell cortical differentiation
Source: Nucleic Acids Res. 2021 Feb 5;49(4):1935–50. doi: 10.1093/nar/gkab030 (PMC7913681; doi:10.1093/nar/gkab030)
Supplement: gkab030_Supplemental_File [file gkab030_supplemental_file.pdf]

Supplemental information:

PAUPAR and PAX6 Sequentially Regulate Human Embryonic Stem Cell Cortical Differentiation

Yanxin Xu[1,5], Jiajie Xi[1,5], Guiying Wang[1,5], Zhenming Guo[2,3], Qiaoyi Sun[1], Chenqi Lu[4], Li Ma[1], Yukang Wu[1], Wenwen Jia[1], Songcheng Zhu[1], Xudong Guo[1], Shan Bian[2], Jiuhong Kang[1*]

1. Supplemental figures (S1-S7)
2. Supplemental figure legends
3. Supplemental methods and materials
4. Supplemental tables

Figure S1

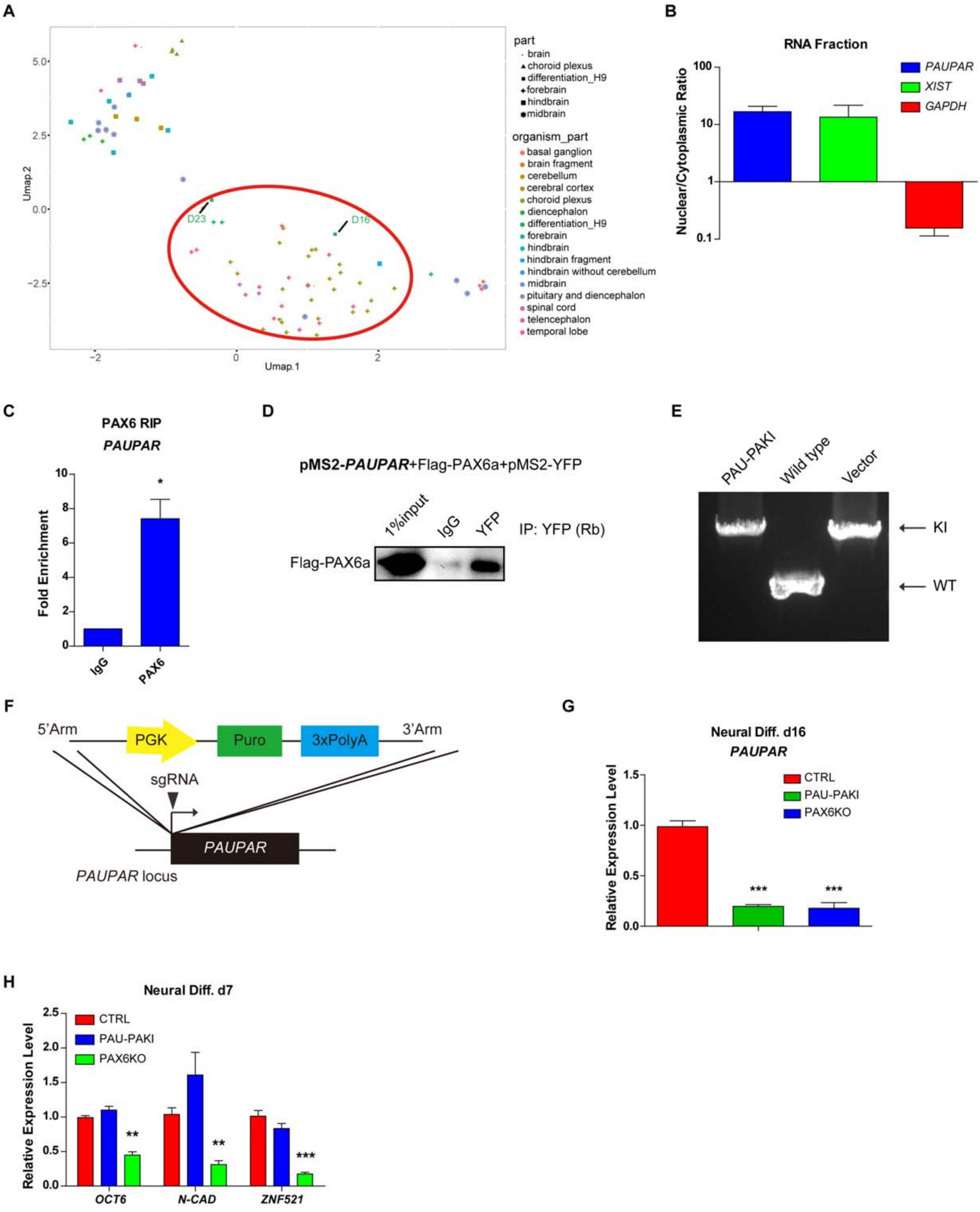

A
part
brain
choroid plexus
differentiation_H9
forebrain
hindbrain
midbrain
organism_part
basal ganglion
brain fragment
cerebellum
cerebral cortex
choroid plexus
diencephalon
differentiation_H9
forebrain
hindbrain
hindbrain fragment
hindbrain without cerebellum
midbrain
pituitary and diencephalon
spinal cord
telencephalon
temporal lobe
D23
D16
Umap.1
Umap.2
B
RNA Fraction
Nuclear/Cytoplasmic Ratio
PAUPAR
XIST
GAPDH
C
PAX6 RIP
PAUPAR
Fold Enrichment
IgG
PAX6
D
pMS2-PAUPAR+Flag-PAX6a+pMS2-YFP
1%input
IgG
YFP
IP: YFP (Rb)
Flag-PAX6a
E
PAU-PAKI
Wild type
Vector
KI
WT
F
5'Arm
PGK
Puro
3xPolyA
3'Arm
sgRNA
PAUPAR
PAUPAR locus
G
Neural Diff. d16
PAUPAR
Relative Expression Level
CTRL
PAU-PAKI
PAX6KO
H
Neural Diff. d7
Relative Expression Level
CTRL
PAU-PAKI
PAX6KO
OCT6
N-CAD
ZNF521

Figure S2

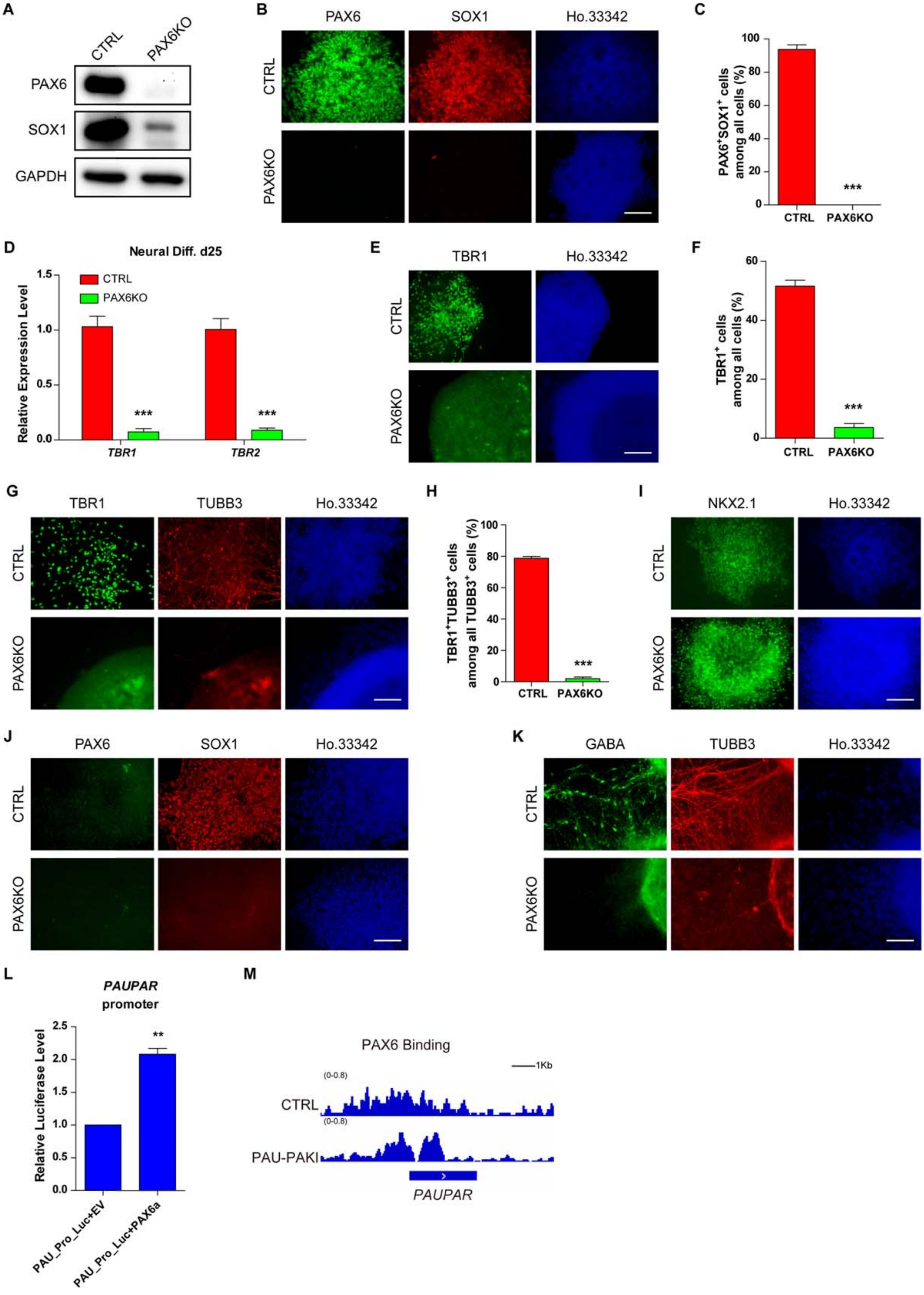

A
CTRL
PAX6KO
PAX6
SOX1
GAPDH
B
PAX6
SOX1
Ho.33342
CTRL
PAX6KO
C
PAX6+SOX1+ cells among all cells (%)
CTRL
PAX6KO
***
D
Neural Diff. d25
CTRL
PAX6KO
Relative Expression Level
TBR1
TBR2
***
E
TBR1
Ho.33342
F
TBR1+ cells among all cells (%)
G
TBR1
TUBB3
Ho.33342
H
TBR1+TUBB3+ cells among all TUBB3+ cells (%)
I
NKX2.1
Ho.33342
J
PAX6
SOX1
Ho.33342
K
GABA
TUBB3
Ho.33342
L
PAUPAR promoter
Relative Luciferase Level
**
PAU_Pro_Luc+EV
PAU_Pro_Luc+PAX6a
M
PAX6 Binding
1Kb
(0-0.8)
CTRL
PAU-PAKI
PAUPAR

Figure S3

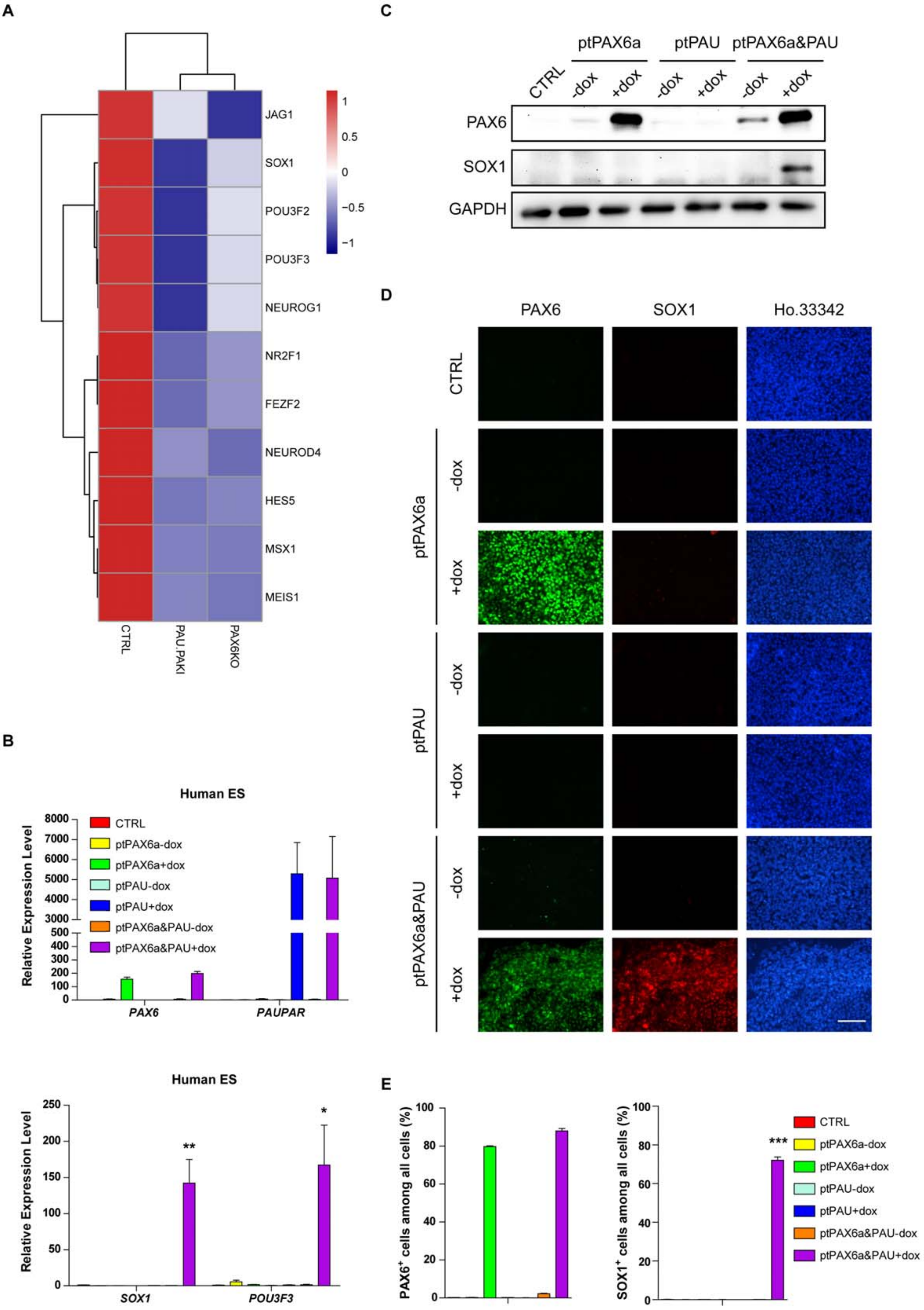
A
JAG1
SOX1
POU3F2
POU3F3
NEUROG1
NR2F1
FEZF2
NEUROD4
HES5
MSX1
MEIS1
CTRL
PAU.PAKI
PAX6KO
1
0.5
0
−0.5
−1
B
Human ES
Relative Expression Level
CTRL
ptPAX6a-dox
ptPAX6a+dox
ptPAU-dox
ptPAU+dox
ptPAX6a&PAU-dox
ptPAX6a&PAU+dox
PAX6
PAUPAR
Human ES
Relative Expression Level
SOX1
POU3F3
**
*
C
ptPAX6a
ptPAU
ptPAX6a&PAU
CTRL
-dox
+dox
PAX6
SOX1
GAPDH
D
PAX6
SOX1
Ho.33342
CTRL
ptPAX6a
ptPAU
ptPAX6a&PAU
-dox
+dox
E
PAX6+ cells among all cells (%)
SOX1+ cells among all cells (%)
***
CTRL
ptPAX6a-dox
ptPAX6a+dox
ptPAU-dox
ptPAU+dox
ptPAX6a&PAU-dox
ptPAX6a&PAU+dox

Figure S4

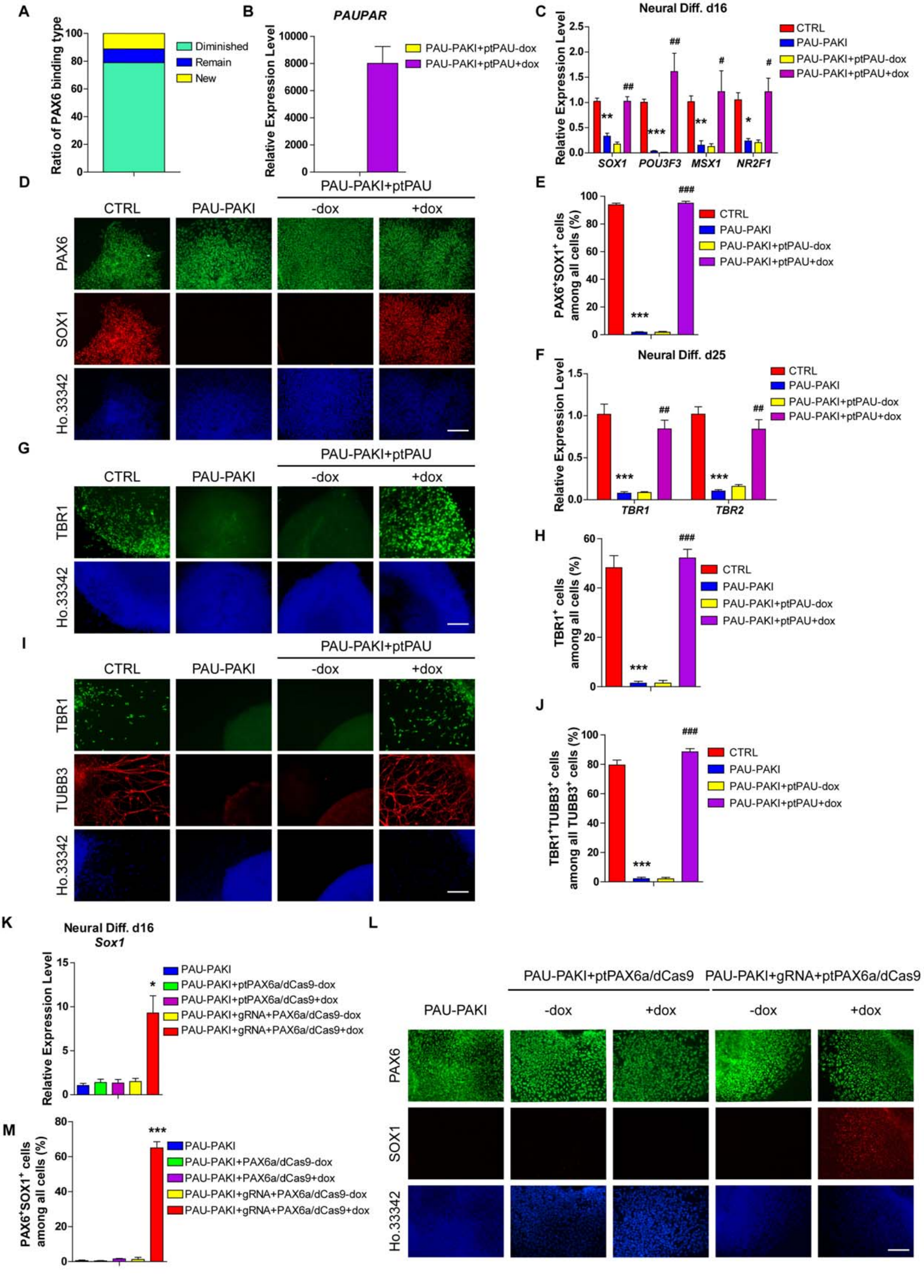

A
Ratio of PAX6 binding type
Diminished
Remain
New
B
PAUPAR
Relative Expression Level
PAU-PAKI+ptPAU-dox
PAU-PAKI+ptPAU+dox
C
Neural Diff. d16
CTRL
PAU-PAKI
SOX1
POU3F3
MSX1
NR2F1
D
PAU-PAKI+ptPAU
-dox
+dox
PAX6
SOX1
Ho.33342
E
PAX6+SOX1+ cells among all cells (%)
F
Neural Diff. d25
TBR1
TBR2
G
H
TBR1+ cells among all cells (%)
I
TUBB3
J
TBR1+TUBB3+ cells among all TUBB3+ cells (%)
K
Sox1
PAU-PAKI+ptPAX6a/dCas9-dox
PAU-PAKI+ptPAX6a/dCas9+dox
PAU-PAKI+gRNA+PAX6a/dCas9-dox
PAU-PAKI+gRNA+PAX6a/dCas9+dox
L
PAU-PAKI+ptPAX6a/dCas9
PAU-PAKI+gRNA+ptPAX6a/dCas9
M
PAU-PAKI+PAX6a/dCas9-dox
PAU-PAKI+PAX6a/dCas9+dox

Figure S5

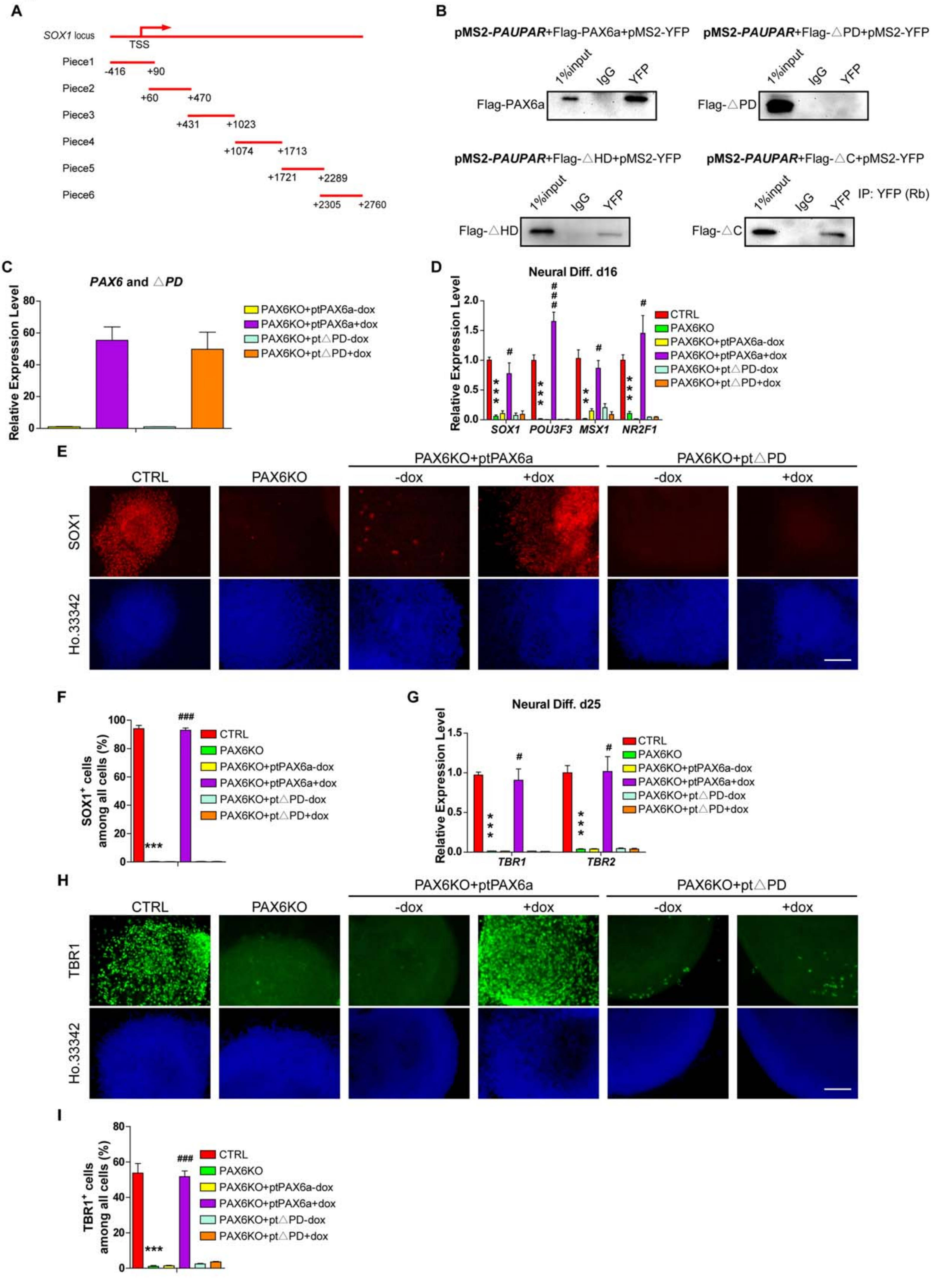
A
SOX1 locus
TSS
Piece1
-416
+90
Piece2
+60
+470
Piece3
+431
+1023
Piece4
+1074
+1713
Piece5
+1721
+2289
Piece6
+2305
+2760
B
pMS2-PAUPAR+Flag-PAX6a+pMS2-YFP
pMS2-PAUPAR+Flag-△PD+pMS2-YFP
pMS2-PAUPAR+Flag-△HD+pMS2-YFP
pMS2-PAUPAR+Flag-△C+pMS2-YFP
1%input
IgG
YFP
Flag-PAX6a
Flag-△PD
Flag-△HD
Flag-△C
IP: YFP (Rb)
C
PAX6 and △PD
Relative Expression Level
PAX6KO+ptPAX6a-dox
PAX6KO+ptPAX6a+dox
PAX6KO+pt△PD-dox
PAX6KO+pt△PD+dox
D
Neural Diff. d16
Relative Expression Level
CTRL
PAX6KO
PAX6KO+ptPAX6a-dox
PAX6KO+ptPAX6a+dox
PAX6KO+pt△PD-dox
PAX6KO+pt△PD+dox
SOX1
POU3F3
MSX1
NR2F1
E
PAX6KO+ptPAX6a
PAX6KO+pt△PD
CTRL
PAX6KO
-dox
+dox
SOX1
Ho.33342
F
SOX1+ cells among all cells (%)
***
###
G
Neural Diff. d25
Relative Expression Level
TBR1
TBR2
H
PAX6KO+ptPAX6a
PAX6KO+pt△PD
CTRL
PAX6KO
-dox
+dox
TBR1
Ho.33342
I
TBR1+ cells among all cells (%)
***
###

Figure S6

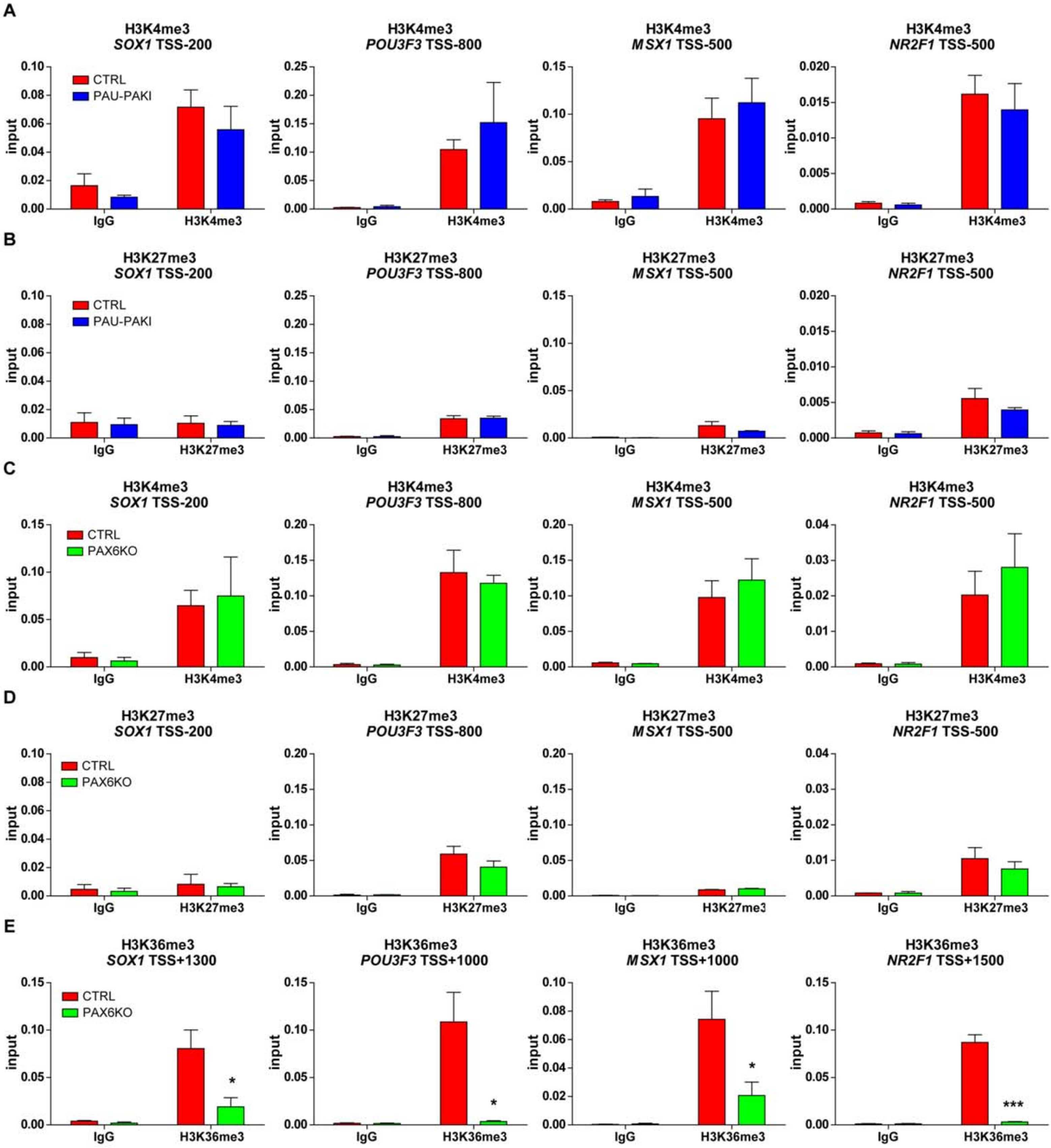

A
H3K4me3
SOX1 TSS-200
H3K4me3
POU3F3 TSS-800
H3K4me3
MSX1 TSS-500
H3K4me3
NR2F1 TSS-500
CTRL
PAU-PAKI
input
IgG
H3K4me3
B
H3K27me3
SOX1 TSS-200
H3K27me3
POU3F3 TSS-800
H3K27me3
MSX1 TSS-500
H3K27me3
NR2F1 TSS-500
CTRL
PAU-PAKI
input
IgG
H3K27me3
C
H3K4me3
SOX1 TSS-200
H3K4me3
POU3F3 TSS-800
H3K4me3
MSX1 TSS-500
H3K4me3
NR2F1 TSS-500
CTRL
PAX6KO
input
IgG
H3K4me3
D
H3K27me3
SOX1 TSS-200
H3K27me3
POU3F3 TSS-800
H3K27me3
MSX1 TSS-500
H3K27me3
NR2F1 TSS-500
CTRL
PAX6KO
input
IgG
H3K27me3
E
H3K36me3
SOX1 TSS+1300
H3K36me3
POU3F3 TSS+1000
H3K36me3
MSX1 TSS+1000
H3K36me3
NR2F1 TSS+1500
CTRL
PAX6KO
input
IgG
H3K36me3
*
***

Figure S7

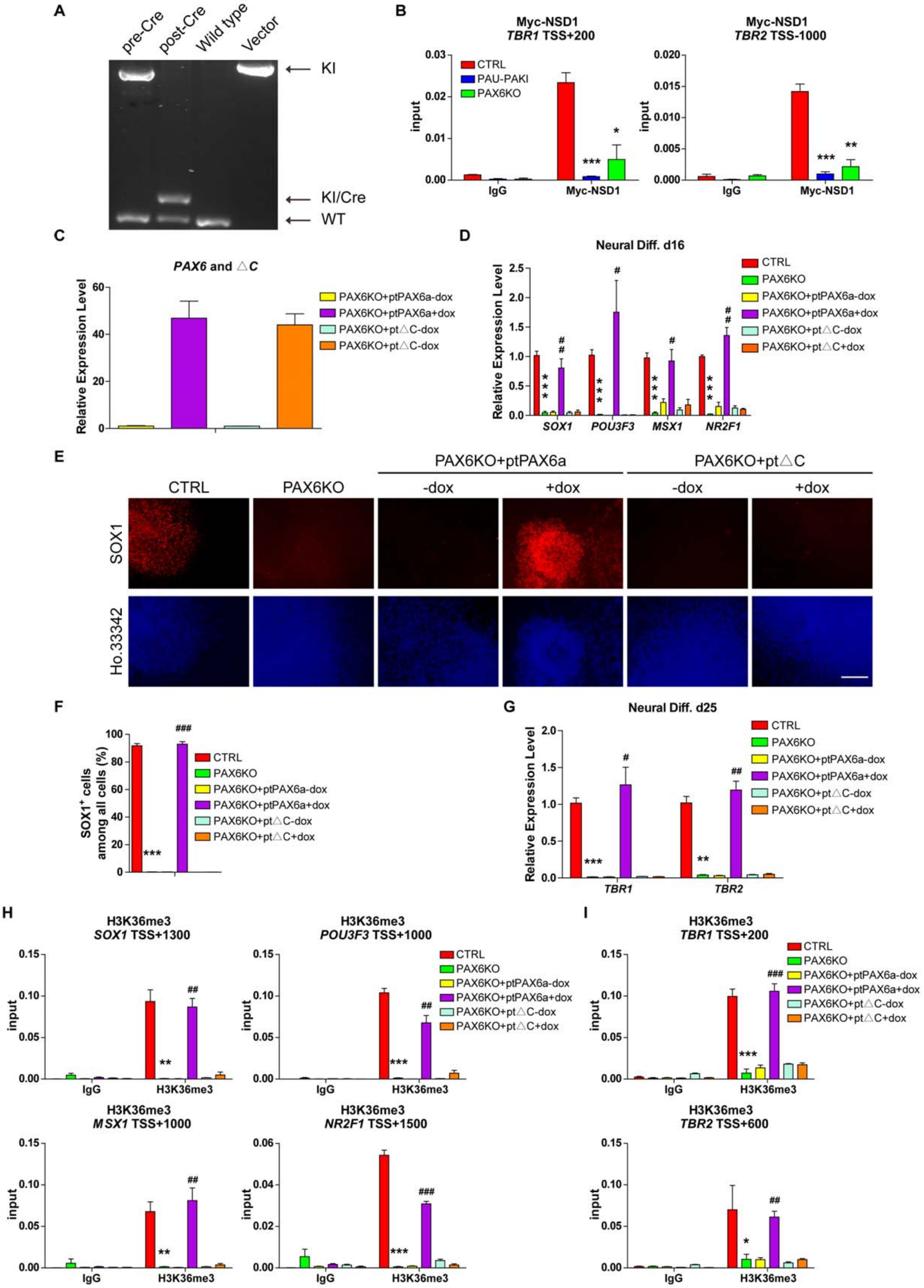
A
pre-Cre
post-Cre
Wild type
Vector
KI
KI/Cre
WT
B
Myc-NSD1
TBR1 TSS+200
Myc-NSD1
TBR2 TSS-1000
CTRL
PAU-PAKI
PAX6KO
input
IgG
Myc-NSD1
C
PAX6 and △C
Relative Expression Level
PAX6KO+ptPAX6a-dox
PAX6KO+ptPAX6a+dox
PAX6KO+pt△C-dox
PAX6KO+pt△C-dox
D
Neural Diff. d16
CTRL
PAX6KO
PAX6KO+ptPAX6a-dox
PAX6KO+ptPAX6a+dox
PAX6KO+pt△C-dox
PAX6KO+pt△C+dox
SOX1
POU3F3
MSX1
NR2F1
E
PAX6KO+ptPAX6a
PAX6KO+pt△C
CTRL
PAX6KO
-dox
+dox
SOX1
Ho.33342
F
SOX1+ cells among all cells (%)
G
Neural Diff. d25
TBR1
TBR2
H
H3K36me3
SOX1 TSS+1300
POU3F3 TSS+1000
MSX1 TSS+1000
NR2F1 TSS+1500
I
TBR1 TSS+200
TBR2 TSS+600
IgG
H3K36me3

Figure S1. *PAUPAR* is a PAX6 binding RNA. Related to Figure 1.

(A) Uniform manifold approximation and projection (UMAP) plot of hESCs dorsal neural differentiated on day 16 and day 23 with tissues from 12 weeks human fetal brain. Colors and forms denote cells sampled from differentiation tissues.

(B) QRT-PCR analysis of RNAs derived from the cytosolic or nuclear fractions on d16 of dorsal neural differentiation. The *XIST* RNA and *GAPDH* mRNA were considered as the positive controls for the nuclear and cytoplasmic fractions, respectively.

(C) RIP analysis for endogenous interaction between PAX6 and *PAUPAR* on day 16 of dorsal neural differentiation.

(D) MS2bp-YFP RNA pull-down analysis for the interaction between Flag-PAX6a and *PAUPAR* in 293FT extracts.

(E) Analyses of the genetic integration of 3×PolyA insertion into the start site of *PAUPAR*.

(F) Schematic representation of the strategy in construction of PAU-PAKI cell line.

(G) Expression of *PAUPAR* in control, PAU-PAKI and PAX6KO cells on day 16 of dorsal neural differentiation.

(H) Expression of *OCT6*, *N-CAD* and *ZNF521* in control, PAU-PAKI and PAX6KO cells on day 7 of neural differentiation.

Data are presented as mean ±SEM. **$p<0.01$, ***$p<0.001$ (t-test).

Figure S2. PAX6 knockout inhibits both dorsal and ventral telencephalon differentiation and *PAX6* regulates *PAUPAR* expression. Related to Figure 1.

(A) Protein level of PAX6 and SOX1 in control and PAX6KO cells on day 16 of dorsal neural differentiation.

(B and C) Immunostaining assay of PAX6 (green) and SOX1 (red) in control and PAX6KO cells on day 16 of dorsal neural differentiation (B), and quantification of PAX6$^+$SOX1$^+$ cells (C). Scale bar, 100 μm.

(D) Expression of *TBR1* and *TBR2* in control and PAX6KO cells on day 25 of dorsal neural differentiation.

(E and F) Immunostaining assay of TBR1 (green) in control and PAX6KO cells on day 25 of dorsal neural differentiation (E), and quantification of TBR1$^+$ cells (F). Scale bar, 100 μm.

(G and H) Immunostaining assay of TBR1 (green) and TUBB3 (red) in control and PAX6KO cells on day 30 of dorsal neural differentiation (G), and quantification of TBR1$^+$TUBB3$^+$ cells among all TUBB3$^+$ cells (H). Scale bar, 100 μm.

(I) Immunostaining assay of NKX2.1 (green) in control and PAX6KO cells on day 25 of ventral neural differentiation. Scale bar, 100 μm.

(J) Immunostaining assay of PAX6 (green) and SOX1 (red) in control and PAX6KO cells on day 16 of ventral neural differentiation. Scale bar, 100 μm.

(K) Immunostaining assay of GABA (green) and TUBB3 (red) in control and PAX6KO cells on day 30 of ventral neural differentiation. Scale bar, 100 μm.

(L) Luciferase activity reporter assay for the *PAUPAR* promoter co-transfected with PAX6a or control vector.

(M) Genome browser screenshots of PAX6 ChIP-seq on *PAUPAR* in control and PAU-PAKI cells on day 16 of dorsal neural differentiation.

Data are presented as mean ±SEM. *$p<0.05$, **$p<0.01$, ***$p<0.001$ (t-test).

Figure S3. *PAUPAR* and *PAX6* can directly promote downstream neural gene expression. Related to Figure 3.

(A) Heatmap of representative downregulated genes related to neural differentiation (e.g., *SOX1*, *POU3F2*, *POU3F3*, *NR2F1* and *MSX1*) in control, PAU-PAKI and PAX6KO cells on day 16 of dorsal neural differentiation.

(B) Expression of *PAX6*, *PAUPAR* (up) and *SOX1*, *POU3F3* (down) in control, PAX6a overexpression, *PAUPAR* overexpression or PAX6a/*PAUPAR* co-overexpression hESCs.

(C) Protein level of PAX6 and SOX1 in control, PAX6a overexpression, *PAUPAR* overexpression or PAX6a/*PAUPAR* co-overexpression hESCs.

(D and E) Immunostaining assay of PAX6 (green) and SOX1 (red) in control, PAX6a overexpression, *PAUPAR* overexpression or PAX6a/*PAUPAR* co-overexpression hESCs (D), and quantification of $PAX6^+$ (left) and $SOX1^+$ (right) cells (E). Scale bar, 100 μm.

Data are presented as mean ±SEM. *p<0.05, **p<0.01, ***p<0.001 (t-test).

Figure S4. *PAUPAR* overexpression can rescue the defects of dorsal telencephalon caused by inhibition of *PAUPAR*. Related to Figure 4.

(A) ChIP-seq analysis showed the fractions of PAX6 binding genes diminished (green), remaining (blue), and new generated (yellow) found after the inhibition of *PAUPAR* on day 16 of dorsal neural differentiation.

(B) QPCR analysis showed the overexpression of *PAUPAR* in the PAU-PAKI cells

(C) Expression of *SOX1*, *POU3F3*, *MSX1* and *NR2F1* after *PAUPAR* overexpression in the PAU-PAKI cells on day 16 of dorsal neural differentiation.

(D and E) Immunostaining assay of PAX6 (green) and SOX1 (red) after *PAUPAR* overexpression in the PAU-PAKI cells on day 16 of dorsal neural differentiation (D), and quantification of $PAX6^+SOX1^+$ cells (E). Scale bar, 100 μm.

(F) Expression of *TBR1* and T*BR2* after *PAUPAR* overexpression in the PAU-PAKI cells on day 25 of dorsal neural differentiation.

(G and H) Immunostaining assay of TBR1 (green) after *PAUPAR* overexpression in the PAU-PAKI cells on day 25 of dorsal neural differentiation (G), and quantification of $TBR1^+$ cells (H). Scale bar, 100 μm.

(I and J) Immunostaining assay of TBR1 (green) and TUBB3 (red) after *PAUPAR* overexpression in the PAU-PAKI cells on day 30 of dorsal neural differentiation (I), and quantification of $TBR1^+TUBB3^+$ cells among all $TUBB3^+$ cells (J). Scale bar, 100 μm.

(K) Expression of *SOX1* after PAX6a/dCas9 overexpression w/o sgRNA in the PAU-PAKI cells on day 16 of dorsal neural differentiation.

(L and M) Immunostaining assay of PAX6 (green) and SOX1 (red) after PAX6a/dCas9 overexpression w/o sgRNA in the PAU-PAKI cells on day 16 of dorsal neural differentiation (L) and quantification of $PAX6^+SOX1^+$ cells (M). Scale bar, 100 μm.

Data are presented as mean ±SEM. *p<0.05, **p<0.01, ***p<0.001 versus CTRL group (C, E, F, H and J), or versus PAU-PAKI+gRNA+ptPAX6a/dCas9-dox group (K and M); #p<0.05, ##p<0.01, ###p<0.001 versus PAU-PAKI+ptPAU-dox group (C, E, F, H and J); (t-test/ANOVA).

Figure S5. PD domain of PAX6 is responsible for binding with *PAUPAR*. Related to Figure 5.

(A) Schematic representation of *SOX1* pieces.

(B) MS2bp-YFP RNA pulldown analysis for the interaction of co-transfected *PAUPAR* with PAX6a, ΔPD, ΔHD and ΔC mutant in 293FT extracts.

(C) QRT-PCR analysis showed the overexpression of *PAX6a* and ΔPD mutant in the PAX6KO cells.

(D) Expression of *SOX1*, *POU3F3*, *MSX1* and *NR2F1* after *PAX6a* or ΔPD mutant overexpression in the PAX6KO cells on day 16 of dorsal neural differentiation.

(E and F) Immunostaining assay of SOX1 (red) after *PAX6a* or ΔPD mutant overexpression in the PAX6KO cells on day 16 of dorsal neural differentiation (E), and quantification of $SOX1^+$ cells (F). Scale bar, 100 μm.

(G) Expression of *TBR1* and *TBR2* after *PAX6a* or ΔPD mutant overexpression in the PAX6KO cells on day 25 of dorsal neural differentiation.

(H and I) Immunostaining assay of TBR1 (green) after *PAX6a* or ΔPD mutant overexpression in the PAX6KO cells on day 25 of dorsal neural differentiation (H), and quantification of $TBR1^+$ cells (I). Scale bar, 100 μm.

Data are presented as mean ±SEM. */p<0.05, **p<0.01, ***/p<0.001 versus CTRL group (D, F, G and I); #/p<0.05, ###/p<0.001 versus PAX6KO+ptPAX6a-dox group (D, F, G and I); (t-test/ANOVA).

Figure S6. *PAX6* knockout does not affect H3K4me3 and H3K27me3 but reduce H3K36me3 level of target gene. Related to Figure 6.

(A and B) Enrichment of H3K4me3 (A) and H3K27me3 (B) on *SOX1*, (TSS -200), *POU3F3* (TSS -800), *MSX1* (TSS -500) and *NR2F1* (TSS -500) genomic region in control and PAU-PAKI cells on day 16 of dorsal neural differentiation.

(C and D) Enrichment of H3K4me3 (C) and H3K27me3 (D) on *SOX1*, (TSS -200), *POU3F3* (TSS -800), *MSX1* (TSS -500) and *NR2F1* (TSS -500) genomic region in control and PAX6KO cells on day 16 of dorsal neural differentiation.

(E) Enrichment of H3K36me3 on *SOX1* (TSS +1300), *POU3F3* (TSS +1000), *MSX1* (TSS +1000) and *NR2F1* (TSS +1500) genomic region in control and PAX6KO cells on day 16 of dorsal neural differentiation.

Data are presented as mean ±SEM. *p<0.05, ***p<0.001 (t-test).

Figure S7. C terminus of PAX6 is responsible for binding with NSD1. Related to Figure 7.

(A) Analyses of the genetic integration of 3×myc-tag insertion into the C-terminal of NSD1.

(B) Enrichment of myc-NSD1 on *TBR1* (TSS +200) and *TBR2* (TSS -1000) genomic region in control, PAU-PAKI and PAX6KO cells on day 25 of dorsal neural differentiation.

(C) QRT-PCR analysis showed the overexpression of *PAX6a* and ΔC mutant in the PAX6KO cells.

(D) Expression of *SOX1*, *POU3F3*, *MSX1* and *NR2F1* after *PAX6a* or ΔC mutant overexpression in the PAX6KO cells on day 16 of dorsal neural differentiation.

(E and F) Immunostaining assay of SOX1 (red) after *PAX6a* or ΔC mutant overexpression in the PAX6KO cells on day 16 of dorsal neural differentiation (E), and quantification of $SOX1^+$

cells (F). Scale bar, 100 μm.

(G) Expression of *TBR1* and *TBR2* after *PAX6a* or ΔC mutant overexpression in the PAX6KO cells on day 25 of dorsal neural differentiation.

(H and I) Enrichment of H3K36me3 on *SOX1* (TSS +1300), *POU3F3* (TSS +1000), *MSX1* (TSS +1000) and *NR2F1* (TSS +1500) genomic region after *PAX6a* or ΔC mutant overexpression in the PAX6KO cells on day 16 (H), *TBR1* (TSS +200) and *TBR2* (TSS +600) genomic region on day 25 (I) of dorsal neural differentiation.

Data are presented as mean ±SEM. *p<0.05, **p<0.01, ***p<0.001 versus CTRL group (B, D and F-I); #p<0.05, ##p<0.01, ###p<0.001 versus PAX6KO+ptPAX6a-dox group (D and F-I); (t-test/ANOVA).

Supplemental methods and materials

hESC electroporation

hESCs were pretreated with 10 μM Y27632 for at least 12 h. Then, the cells were digested into single cells with Accutase and electroporated with plasmids. For the CRISPR/Cas9 system, 5 μg of Cas9, 5 μg of gRNA and 20 μg of targeting donor plasmid were used. For the TALEN system, 5 μg of Talen-Left, 5 μg of Talen-Right and 20 μg of donor plasmid were used. P3 primary cell buffer (Lonza) was used for each transfection of hESCs with Program CB-150. Transfected cells were plated on an irradiated MEF layer supplied with Y27632 and cultured overnight. The medium was changed every day, and individual colonies were selected with puromycin (1 μg/ml), blasticidin (2 μg/ml) or G418 (200 μg/ml), respectively.

Generation of gene knock-in hESCs by CRISPR/Cas9

To generate PAU-PAKI hESC lines, gRNA targeting PAUPAR listed in Supplemental Table S2 was designed using the CRISPR DESIGN website (http://crispr.mit.edu/). The PGK promoter-puro segment and 3×polyA segment were cloned into the pLB vector (TIANGEN) and integrated into the transcription start site (TSS) of PAUPAR with the designed sgRNA respectively.

To generate NSD1-3×myc hESC lines, gRNA targeting the C-terminus of NSD1 (Supplemental Table S2) was used. The 3×myc tag segment and a stop codon with an EF1A1 promoter-driven neomycin cassette segmented between two LoxP sequences were cloned into the pLB vector and integrated into the end of NSD1. After individual colonies were selected by G418, the Cre recombinase driven by the EF1α promoter was transfected into hESCs through electroporation.

Generation of gene inducible overexpression hESCs by TALEN

To generate gene-inducible overexpression hESC lines, PAUPAR, mutant PAUPAR (missing nt 1-919), wild type PAX6a, PAX6a-ΔPD (with amino acids 2-128 deleted) and PAX6a-ΔC (with amino acids 303-422 deleted) driven by the tetracycline response element (pTREtight promoter, pt) and rtTA driven by the CAG promoter were integrated into the AAVS1 locus through electroporation with TALEN as previously described(1).

To generate inducible PAX6a/dCas9-overexpression hESCs lines, the PAX6a/dCas9 fusion protein

driven by the pTREtight promoter and rtTA driven by the CAG promoter were integrated into one allele of the AAVS1 locus, and gRNA targeting the *SOX1* locus (Supplemental Table S2) modified as previously described(2) and driven by the U6 promoter was integrated into another allele through electroporation with TALEN.

Western blotting

The cells were collected, washed with PBS, and then lysed in 1× SDS lysis buffer with protease inhibitor cocktail (Roche). Lysates were separated from cell extracts by 10-12% Bis-Tris SDS-PAGE and transferred to polyvinylidene difluoride (PVDF) membranes (Millipore). Blots were blocked in 3% BSA/TBST at room temperature for 1 h and incubated with primary antibody at 4°C overnight and secondary antibody at room temperature for 1 h. The signals were visualized by using enhanced chemiluminescence (ECL). The antibodies used in this study are listed in Supplemental Table S1.

Quantitative RT-PCR

Total RNA was extracted from the cells using RNAiso (Takara) according to the manufacturer's instructions. A total of 500 ng of total RNA was reverse transcribed into cDNA using a PrimeScript™ RT reagent kit (Takara). The resultant cDNA was used for QRT-PCR on an Mx3000 instrument (Agilent) with relative expression levels calculated using the $2^{-\Delta\Delta Ct}$ method(3) by normalizing against *GAPDH* expression; results are presented as the fold change relative to the control. Each experiment was performed in triplicate and repeated three times. The primer sequences used in this study are listed in Supplemental Table S2.

Fluorescent *in situ* hybridization (FISH)

FISH assays were performed as previously described (4). Cells cultured on coverslips were fixed with 4% paraformaldehyde at 4°C for 15 min and washed three times with PBS. The samples were subsequently incubated with Pre-Hybridization Buffer at 37℃ for 30 min and Hybridization Buffer with FISH probes at 37°C overnight in the dark using a Ribo™ Fluorescent *In Situ* Hybridization Kit

(RiboBio). The next day, the coverslips were washed three times with wash buffer I (4× SSC with 0.1% Tween-20), once each with wash buffer II (2× SSC) and wash buffer III (1× SSC) at 42°C in the dark for 5 min and once with PBS at room temperature. Then, the cells were stained with Ho 33342 in the dark for 10 min. PAUPAR-Cy3 FISH probes (RiboBio) were designed and synthesized by RiboBio Co., Ltd. Human U6 FISH probes (RiboBio) and Human 18S FISH probes (RiboBio) were used as nuclear and cytoplasmic controls, respectively. All images were captured by fluorescence microscopy (Nikon).

Luciferase reporter assay

Luciferase activities were examined using the Dual-Luciferase Reporter Assay System (Promega) as described previously (5). Luciferase reporters were generated by cloning the PAUPAR promoter (-2000 to +1000) into the pGL3-Basic vector (Promega). Then, 293FT cells (5×10^4) were plated in 24-well plates, and the cells in each well were transfected with luciferase reporters (100 ng), Renilla (5 ng) and PAX6a (pcDNA3.1 vector, 150 ng). Cell lysates were harvested 48 h after transfection.

Chromatin isolation by RNA purification (ChIRP)

ChIRP was performed as previously described with modifications (6,7). Antisense probes were designed using an online probe designer (singlemoleculefish.com). Cells were crosslinked with 1% glutaraldehyde (Sangon Biotech) for 10 min and quenched with 0.25 M glycine for 5 min at room temperature. The cell pellet was lysed in nuclei lysis buffer (50 mM Tris (pH 7.0), 10 mM EDTA and 1% SDS) with the addition of dithiothreitol, protease inhibitor cocktail, PMSF and RNaseOUT (Invitrogen). Then, each sample was sonicated to generate 100-500 bp fragments. Chromatin fractions were diluted in two volumes of hybridization buffer (50 mM Tris (pH 7.0), 750 mM NaCl, 0.1% SDS, 1 mM EDTA, 1% Triton X-100 and 15% formamide (Sigma) with dithiothreitol, protease inhibitor cocktail, PMSF and RNaseOUT) supplemented with 100 pmol of probes by end-to-end rotation at 37°C for 4 h. Then, 100 µl of prewashed streptavidin magnetic C1 beads (invitrogen) was added and incubated at 37°C by end-to-end rotation for an additional 0.5-1 h. The beads were then washed five times with wash buffer (2× SSC and 0.1% SDS supplemented with dithiothreitol

and fresh PMSF). For RNA elution, beads were resuspended in RNA pK buffer (10 mM Tris (pH 7.0), 100 mM NaCl, 1 mM EDTA, 0.5% SDS) and Proteinase K. Then, samples were incubated at 65°C for 45 min with end-to-end shaking, followed by boiling at 95°C for 10 min. Immunoprecipitated RNAs were extracted using RNAiso (Takara) and analyzed by QRT-PCR. For DNA elution, beads were washed once with SDS elution buffer (50 mM Tris, 75 mM NaCl, 5 mM $MgCl_2$, 1% SDS) at 37°C for 30 min, followed by one wash with elution buffer (50 mM Tris, 75 mM NaCl, 5 mM $MgCl_2$, 0.1% Triton X-100) at 37°C for 5 min. Then, DNA was sequentially eluted by elution buffer supplemented with RNaseH and RNaseA and by SDS elution buffer at room temperature for 2 min. The combined eluents were treated with proteinase K, 150 mM NaCl and 10 mM EDTA at 65°C for 50 min. DNA was then purified with phenol-chloroform (Sangon Biotech) and subjected to QRT-PCR analysis. The primer sequences and the probe sequences used in this study are listed in Supplemental Table S2.

Coimmunoprecipitation (Co-IP)

Co-IP assays were performed as previously described with modifications (8). A mixture of Ezview Red Protein A affinity gel (Sigma) and Ezview Red Protein G affinity gel (Sigma) (1:1) was incubated with antibodies or control normal IgG overnight at 4°C. Cells were lysed in RIPA buffer containing a protease inhibitor cocktail (Roche) on ice. The cells were sonicated for 30 s (2 s interval) at 25 Amps (Qsonica Sonicators), after which antibodies were added and incubated for 4-6 h at 4°C. The protein-bead complex was washed with RIPA buffer 3 times and subjected to western blotting analysis. The antibodies used in this study are listed in Supplemental Table S1.

MS2bp-YFP RNA pulldown

The MS2bp-YFP RNA pulldown assay was performed as described previously(9). A total of 5×10^6 293FT cells were seeded into 100-mm plates and cotransfected with 4 µg of MS2bs overexpression vector (pcDNA3-MS2bs), 4 µg of the MS2bp-YFP overexpression plasmid, and 4 µg of pcDNA overexpression vector with 36 µl of FuGENE HD (Roche). After 48 h, the cells were lysed with RIP buffer (100 mM KCl, 5 mM MgCl2, 10 mM HEPES (pH 7.0), 0.5% NP-40, and 1 mM dithiothreitol)

for 30 min on ice. The proteins were immunoprecipitated using control IgG (CST) or anti-GFP antibody (Abcam), which was able to recognize the YFP protein. The RNA and RNA-bound protein complexes were treated with RNAiso (Takara) to purify the RNA or SDS lysis buffer for western blotting analysis.

Chromatin immunoprecipitation (ChIP)

ChIP assay was performed as described previously (4). Cells were crosslinked with 1% formaldehyde (Sigma) for 10 min, and the crosslinking reaction was then quenched with 0.25 M glycine for 5 min at room temperature. Cell pellets were lysed in cell lysis buffer (5 mM PIPES (pH 8pH 8.0), 85 mM KCl and 0.5% NP-40), followed by nuclei lysis buffer (50 mM Tris (pH 8.1), 10 mM EDTA and 0.75% SDS), and sonicated using an M220 Focused-ultrasonicator (Covaris) to generate 500 to 750 bp fragments. Fragmented chromatin samples were incubated with magnetic beads (ChIP-grade Protein G beads; CST) prebound with 4 µg of antibody at 4°C. The beads were washed once with TSE buffer I (20 mM Tris (pH 8.1), 150 mM NaCl, 0.1%SDS, 1% TritonX-100 and 2 mM EDTA), TSE buffer II (20 mM Tris (pH 8.1), 500 mM NaCl, 0.1% SDS, 1% TritonX-100 and 2 mM EDTA), wash buffer III (10 mM Tris (pH 8.1), 250 mM LiCl, 1% deoxycholate, 1% NP-40 and 1 mM EDTA) and TE buffer (10 mM Tris (pH 8.1) and 1 mM EDTA). Each wash was performed on a rotator over 15 min at 4°C. Beads were treated with 300 µl of elution buffer (100 mM NaHCO3 and 1% SDS). 30 µl of 5 M NaCl was added to the eluted samples except input. All samples were reverse crosslinked overnight at 65°C. After RNaseA and proteinase K treatment, samples were purified with phenol-chloroform (Sangon Biotech). Immunoprecipitated DNA and input DNA were used as templates for QRT-PCR analysis. The antibodies used in this study are listed in Supplemental Table S1. The primer sequences used in this study are listed in Supplemental Table S2.

RNA-seq

RNA-Seq library generation and sequencing were performed as previously described (10). Quality control and adapter trimming of sequencing raw data were completed by fastqc (11) and Trim Galore (http://www.bioinformatics.babraham.ac.uk/projects/trim_galore/). All RNA-seq reads were aligned to the human genome (hg19) using TopHat (v2.0.12) with default parameters

(12). Gene expression levels were measured as FPKM using Cufflinks (v2.2.1) to eliminate the effects of sequencing depth and transcript length (13). For each comparison, differentially expressed (DE) genes with a GFOLD value larger than 1 (fold change larger than 2) were found using GFOLD (v1.1.3) (14). For the following analysis, FPKMs were log2 transformed after adding a pseudocount of 1. Functional annotation was performed using the Database for Annotation, Visualization and Integrated Discovery (DAVID 6.7) bioinformatics resource (15). GO terms for each functional cluster were summarized as a representative term, and P-values were plotted to show the significance.

ChIP-seq

ChIP was performed using the SimpleChIP Enzymatic Chromatin IP Kit (Magnetic Beads) (Cell Signaling Technology). DNA libraries were constructed by following Illumina library preparation protocols. All ChIP-seq reads were aligned to the human genome (hg19) using Bowtie2 (v2.2.9) with default parameters (16). The reads signal for each sample were generated using MACS2 (v2.1.1.20160309) and normalized to 1 million reads for visualization with the parameter –SPMR (17). The normalized reads signal were used to calculate the average signal of each sample around the TSS in different groups of genes. The binding motif of PAX6 was predicted by the MEME method (18). The antibodies used in this study are listed in Supplemental Table S1.

References

1. Hockemeyer, D., Wang, H., Kiani, S., Lai, C.S., Gao, Q., Cassady, J.P., Cost, G.J., Zhang, L., Santiago, Y., Miller, J.C. *et al.* (2011) Genetic engineering of human pluripotent cells using TALE nucleases. *Nature biotechnology*, **29**, 731-734.
2. Zalatan, J.G., Lee, M.E., Almeida, R., Gilbert, L.A., Whitehead, E.H., La Russa, M., Tsai, J.C., Weissman, J.S., Dueber, J.E., Qi, L.S. *et al.* (2015) Engineering complex synthetic transcriptional programs with CRISPR RNA scaffolds. *Cell*, **160**, 339-350.
3. Livak, K.J. and Schmittgen, T.D. (2001) Analysis of relative gene expression data using real-time quantitative PCR and the 2(-Delta Delta C(T)) Method. *Methods*, **25**, 402-408.
4. Guo, X., Xu, Y., Wang, Z., Wu, Y., Chen, J., Wang, G., Lu, C., Jia, W., Xi, J., Zhu, S. *et al.* (2018) A Linc1405/Eomes Complex Promotes Cardiac Mesoderm Specification and Cardiogenesis. *Cell stem cell*, **22**, 893-908 e896.
5. Xi, J., Wu, Y., Li, G., Ma, L., Feng, K., Guo, X., Jia, W., Wang, G., Yang, G., Li, P. *et al.* (2017) Mir-29b Mediates the Neural Tube versus Neural Crest Fate Decision during Embryonic Stem Cell

Neural Differentiation. *Stem cell reports*, **9**, 571-586.

6. Chu, C., Qu, K., Zhong, F.L., Artandi, S.E. and Chang, H.Y. (2011) Genomic maps of long noncoding RNA occupancy reveal principles of RNA-chromatin interactions. *Molecular cell*, **44**, 667-678.
7. Trimarchi, T., Bilal, E., Ntziachristos, P., Fabbri, G., Dalla-Favera, R., Tsirigos, A. and Aifantis, I. (2014) Genome-wide mapping and characterization of Notch-regulated long noncoding RNAs in acute leukemia. *Cell*, **158**, 593-606.
8. Yu, Y., Jia, W., Lyu, Y., Su, D., Bai, M., Shen, J., Qiao, J., Han, T., Liu, W., Chen, J. *et al.* (2019) Pwp1 regulates telomere length by stabilizing shelterin complex and maintaining histone H4K20 trimethylation. *Cell discovery*, **5**, 47.
9. Yoon, J.H., Abdelmohsen, K., Srikantan, S., Yang, X., Martindale, J.L., De, S., Huarte, M., Zhan, M., Becker, K.G. and Gorospe, M. (2012) LincRNA-p21 suppresses target mRNA translation. *Molecular cell*, **47**, 648-655.
10. Weng, R., Lu, C., Liu, X., Li, G., Lan, Y., Qiao, J., Bai, M., Wang, Z., Guo, X., Ye, D. *et al.* (2018) Long Noncoding RNA-1604 Orchestrates Neural Differentiation through the miR-200c/ZEB Axis. *Stem cells*, **36**, 325-336.
11. Brown, J., Pirrung, M. and McCue, L.A. (2017) FQC Dashboard: integrates FastQC results into a web-based, interactive, and extensible FASTQ quality control tool. *Bioinformatics*, **33**, 3137-3139.
12. Trapnell, C., Pachter, L. and Salzberg, S.L. (2009) TopHat: discovering splice junctions with RNA-Seq. *Bioinformatics*, **25**, 1105-1111.
13. Trapnell, C., Williams, B.A., Pertea, G., Mortazavi, A., Kwan, G., van Baren, M.J., Salzberg, S.L., Wold, B.J. and Pachter, L. (2010) Transcript assembly and quantification by RNA-Seq reveals unannotated transcripts and isoform switching during cell differentiation. *Nature biotechnology*, **28**, 511-515.
14. Feng, J., Meyer, C.A., Wang, Q., Liu, J.S., Shirley Liu, X. and Zhang, Y. (2012) GFOLD: a generalized fold change for ranking differentially expressed genes from RNA-seq data. *Bioinformatics*, **28**, 2782-2788.
15. Huang da, W., Sherman, B.T. and Lempicki, R.A. (2009) Systematic and integrative analysis of large gene lists using DAVID bioinformatics resources. *Nature protocols*, **4**, 44-57.
16. Langmead, B. and Salzberg, S.L. (2012) Fast gapped-read alignment with Bowtie 2. *Nature methods*, **9**, 357-359.
17. Zhang, Y., Liu, T., Meyer, C.A., Eeckhoute, J., Johnson, D.S., Bernstein, B.E., Nusbaum, C., Myers, R.M., Brown, M., Li, W. *et al.* (2008) Model-based analysis of ChIP-Seq (MACS). *Genome biology*, **9**, R137.
18. Bailey, T.L., Johnson, J., Grant, C.E. and Noble, W.S. (2015) The MEME Suite. *Nucleic acids research*, **43**, W39-49.

Table S1. Antibodies used for this study.

Antibodies	SOURCE	IDENTIFIER
Rabbit polyclonal to anti-PAX6	Abcam	Cat# ab5790, RRID: AB_305110
Goat polyclonal to anti-SOX1	R&D Systems	Cat# AF3369, RRID: AB_2239879
Rabbit polyclonal to anti-PAX6	BioLegend	Cat# 901301, RRID: AB_2565003
Rabbit polyclonal to anti-TBR1	Abcam	Cat# ab183032
Mouse monoclonal to anti-TUBB3	Sigma-Aldrich	Cat# T5076, RRID: AB_532291
Mouse monoclonal to anti-NKX2.1	Millipore	Cat# MAB5460, RRID: AB_571072
Rabbit polyclonal to anti-GABA	Sigma-Aldrich	Cat# A2052, RRID: AB_477652
Mouse monoclonal to anti-NESTIN	BD Biosciences	Cat# 611658, RRID: AB_399176
Mouse monoclonal to anti-MAP2	Abcam	Cat# ab11267, RRID: AB_297885
Rabbit polyclonal to anti-GAPDH	Bioworld	Cat# AP0063; RRID: AB_2651132
Alexa Fluor 488 Donkey Anti-Mouse	Invitrogen	Cat# A21202; RRID: AB_141607
Alexa Fluor 488 Donkey Anti-Rabbit	Invitrogen	Cat# A21206; RRID: AB_141708
Alexa Fluor 594 Donkey Anti-Mouse	Invitrogen	Cat# A21203; RRID: AB_141633
Alexa Fluor 594 Donkey Anti-Goat	Invitrogen	Cat# A-11058, RRID: AB_142540
Rabbit polyclonal to anti-GFP	Abcam	Cat# ab290; RRID: AB_2313768
Mouse monoclonal to anti-RNA Polymerase II	Millipore	Cat# 05-623; RRID: AB_309852
Rabbit polyclonal to anti-H3K4me3	Cell Signaling Technology	Cat# 9727, RRID: AB_561095

Mouse monoclonal o anti-H3K27me3	Abcam	Cat# ab6002, RRID: AB_305237
Rabbit polyclonal to anti-H3K36me3	Abcam	Cat# ab9050, RRID: AB_306966
Rabbit polyclonal to anti-H3K36me2	Abcam	Cat# ab9049, RRID: AB_1280939
Mouse monoclonal to anti-Flag	GNI	Cat# GNI4110
Mouse monoclonal to anti-HA-tag	Bioworld	Cat# AP0005M, RRID: AB_2797399
Goat polyclonal to anti-Myc-tag	Abcam	Cat# ab9132, RRID: AB_307033
Mouse monoclonal to anti- Myc-tag	Cell Signaling Technology	Cat# 2276, RRID: AB_331783

Supplemental Tables S2. Primers used for this study.

**Primers used for gRNA.**		
PAUPAR-gRNA	GGACGAAACACCGTAACTCGAACCGGAGAAGCGAGGGTTTTAGAGCTA	F
	TTTCTAGCTCTAAAACCCTCGCTTCTCCGGTTCGAGTTACGGTGTTTC	R
PAX6a/dCAS9-gRNA	GGACGAAACACCGTCAGCGCCCGGCGGTCGCCAGGGGTTTTAGAGCTA	F
	TTTCTAGCTCTAAAACTCAGCGCCCGGCGGTCGCCAGGGCGGTGTTTC	R
NSD1-C-terminal-gRNA	GGACGAAACACCGCATTAGCCAGTGGGGGCTTATGGGTTTTAGAGCTA	F
	TTTCTAGCTCTAAAACCCATAAGCCCCCACTGGCTAATGCGGTGTTTC	R
**Primers used in QRT-PCR of gene expression assays.**		
PAUPAR	GGAGGCAAATAGCGTCAAGGTTTCCTC	F
	CGAATGTTTGTATGCCATATTTGTGC	R
PAX6	TCTTTGCTTGGGAAATCCG	F
	CTGCCCGTTCAACATCCTTAG	R
SOX1	GTTTTTTGTAGTTGTTACCGC	F
	GCATTTACAAGAAATAATAC	R
NKX2.1	AACCAAGCGCATCCAATCTCAAGG	F
	TGTGCCCAGAGTGAAGTTTGGTCT	R
NKX2.2	GTCAGGGACGGCAAACCAT	F
	GCGCTGTAGGCAGAAAAGG	R
TBR1	ACAATTTTCCTGACTCCAAGGAC	F
	ACTGTGACGAAGCTCAGAGAC	R
TBR2	CTGCCCACTACAATGTGTTCG	F
	GCGCCTTTGTTATTGGTGAGTTT	R
OCT6	GCTCGAGAGCCACTTTCTCA	F
	CCAGGCGCGTATACATCGT	R
ZNF521	CAACTGACAGATGGAGTGGATG	F
	GCTAGGGGAAGTCTGATCCTT	R
NCAD	TCCTGATATATGCCCAAGACAA	F
	TGACCCAGTCTCTCTTCTGC	R
GATA4	GTGTCCCAGACGTTCTCAGTC	F
	GGGAGACGCATAGCCTTGT	R
SOX17	GTGGACCGCACGGAATTTG	F
	GGAGATTCACACCGGAGTCA	R
T	AATTGGTCCAGCCTTGGAAT	F

	CGTTGCTCACAGACCACA	R
MIXL1	GGCGTCAGAGTGGGAAATCC	F
	GGCAGGCAGTTCACATCTACC	R
POU3F2	AAGCGGAAAAAGCGGACCT	F
	GTGTGGTGGAGTGTCCCTAC	R
POU3F3	CATTTTTGCAGTCCAAGGAAGGAG	F
	ACACCACCACAGTATGAACGAGATC	R
FEZF2	CGCTGGAGCAGGTACTGAAG	F
	CGGGTGAGATTATAGTGAGCGTT	R
NR2F1	ATCGTGCTGTTCACGTCAGAC	F
	TGGCTCCTCACGTACTCCTC	R
MSX1	ACACAAGACGAACCGTAAGCC	F
	CACATGGGCCGTGTAGAGTC	R
JAG1	GCCGAGGTCCTATACGTTGC	F
	CCGAGTGAGAAGCCTTTTCAA	R
NEUROG1	GCTCTCTGACCCCAGTAGC	F
	GCGTTGTGTGGAGCAAGTC	R
NEUROD4	AAAGCTCGCCTTGAGAGATTC	F
	GCCTCAGGTTATCCAGGGC	R
HES5	TCAGCCCCAAAGAGAAAAAC	F
	TAGTCCTGGTGCAGGCTCTT	R
MEIS1	GATATAGCCGTGTTCGCCAAA	F
	CGGTGGCAGAAATTGTCACAT	R
XIST	AGCTCCTCGGACAGCTGTAA	F
	CTCCAGATAGCTGGCAACC	R
GAPDH	ATGACATCAAGAAGGTGGTG	F
	CATACCAGGAAATGAGCTTG	R
PAX6-C-terminus	GGTGAATGGGCGGAGTTATGAT	F
	GGGGAAATGAGTCCTGTTGAAGTG	R
**Primers used in QRT-PCR of ChIP, ChIRP and in vitro triplex pulldown assays.**		
SOX1-4000	TAATGGTGTTACAGTCCAGCACTCA	F
	GGTCATAATCTTCAAAGCCAATCC	R
SOX1-200	AAGTGGTTTGTGCATCAGGAGAAAC	F
	GAGAGGAATTCAGACGGAGTGAGAC	R
SOX1+300	ATGCACAACTCGGAGATCAGCAAG	F
	GCGAGTACTTGTCCTTCTTGAGCAG	R
SOX1+600	CAAGACCAAGACGCTGCTCAAGAAG	F
	CAGCCGTTGACGTGCGCGTAG	R
SOX1+1300	CTTTTGTACAGACGTTCCCACATTC	F
	CGCTTCCTCCGTAGGTGATAAATAC	R
SOX1+2000	AATGGGAGGACAGGATTTTTCAAGG	F
	CAGTGAAACAGCCGGAGCAGAAG	R

SOX1+2600	ACACACAGAAATATCGACCATCACC	F
	GCATCAAACAACCTCAAGATCTCG	R
POU3F3-4000	ATCAGCACTAACTGTTCTCCCCTTGT	F
	CGTACTTAATTCTCGAATCGCTCCC	R
POU3F3-800	GACTGTTGCGAGGAGAATGAAAAAG	F
	CTAGGAAAACTTCTTGCGATGGTG	R
POU3F3+1000	TTCAAGAACATGTGCAAGCTCAAG	F
	TCACCTCGATAGAGGTCCGCTTC	R
MSX1-4000	TCGGTTTTCATAGCACATTCATTC	F
	TTTTGTTTATGCTCCCTCCCTCT	R
MSX1-500	CTGAAGCACTCCGTGGGCAC	F
	GGAAAGGTGGATGTTAATACAGTTGAC	R
MSX1+1000	AGTTAACGGATAGGACACCGATGTC	F
	TCCTACTGCGAGAAAGTGAAACTCC	R
NR2F1-4000	AATTGAGCCTCTTCCTTTCAACAC	F
	AGCGGATTAAAAGGGACATCG	R
NR2F1-500	GGTCTCAGAGTAAATAGCAGCGCAG	F
	GCTACCTACAGACTGGGATGGTGTG	R
NR2F1+1500	AAGCACTACGGCCAATTCACCT	F
	ATATTCACCTTCCCGCCTCATG	R
TBR1-4000	GCCCCTGTTATTACTTACGGTTCA	F
	CATATTTTCACAAGGGTGCAGACTAG	R
TBR1+200	AAGTGCTTTCTGTCTAGTGAGGGG	F
	TTGTCAGTGGTCGAGATAATGGG	R
TBR2-4000	TGGGAGTATCTTGCTCTTGTCAGC	F
	CACCGTTAGGTTGTTCATGTTTCAT	R
TBR2-1000	CTGAAGATGGTGGTGAAGGGACG	F
	ACAACTGCTTTCGGGGAGACAG	R
TBR2+600	CCAAGAAGTTTTCCGGCAGTC	F
	CGGCGTCGGTGTCACTAAGC	R
GAPDH+3500	ATGACATCAAGAAGGTGGTG	F
	CATACCAGGAAATGAGCTTG	R

ChIRP probes.

PAUPAR-ChIRP-1	AAATTTTTATTCCGGTACCTTT	even
PAUPAR-ChIRP-2	CACCGATTAATTTATCCCAAAC	odd
PAUPAR-ChIRP-3	TTCTTCAAGCCACCGATTAATT	even
PAUPAR-ChIRP-4	CAAAACTTAAATCCTGGCTGGA	odd
PAUPAR-ChIRP-5	CAGCTGCCTCCAAATCAATAGA	even
PAUPAR-ChIRP-6	AGCTAATATATTGTGACTGCTC	odd
PAUPAR-ChIRP-7	TAAAAGTAAGGGTTGAGCCCAA	even
PAUPAR-ChIRP-8	AGTTAAAAGTAAGGGTTGAGCC	odd
PAUPAR-ChIRP-9	CATTAGTAGTACTACTCCATTA	even

PAUPAR-ChIRP-10	GAGTGGAGACTCTTTGGTCTCC	odd
PAUPAR-ChIRP-11	CCACGACCAGCAGAACTTCTCC	even
PAUPAR-ChIRP-12	CTGTTTTCACAATCTGCGTCAC	odd
PAUPAR-ChIRP-13	ATTAGCGAAGGAAATGACCCAA	even
PAUPAR-ChIRP-14	TCAAAGTGAGGCAGACTCTGGC	odd
PAUPAR-ChIRP-15	ATATTTTCTCCATAAAGTGCAG	even
PAUPAR-ChIRP-16	TTTAATTGTCCATGCGTGTTCC	odd
PAUPAR-ChIRP-17	TAAGTGGGTGTGTTTAATTGTC	even
PAUPAR-ChIRP-18	TATCCATTTCTGAACACATGTG	odd
PAUPAR-ChIRP-19	TATAGCAAGGTGCATTAGCTGC	even
PAUPAR-ChIRP-20	AACGGACGTACATCTATAGCAA	odd
PAUPAR-ChIRP-21	CGGCAACAAGAAAAGGTGTCAG	even
PAUPAR-ChIRP-22	CTTCCCCGGCAACAAGAAAAGG	odd
PAUPAR-ChIRP-23	CTGCAGAGGTCTAGTCAAAATA	even
PAUPAR-ChIRP-24	CCCCTGCAGAGGTCTAGTCAAA	odd
PAUPAR-ChIRP-25	AACTACAGTATTCCACAGATGA	even
PAUPAR-ChIRP-26	CAGCAAGACAGACTCTTTAACA	odd
PAUPAR-ChIRP-27	ACTAGTTCTATGGGTTTCCTCA	even
PAUPAR-ChIRP-28	GGAAAGTAGCAGTGACAGAACA	odd
PAUPAR-ChIRP-29	ACGATCGGAAAGTAGCAGTGAC	even
PAUPAR-ChIRP-30	CAGGGAGGGGCGTTAAGAAGAG	odd
PAUPAR-ChIRP-31	CCGAGGAAACCTTGACGCTATT	even
PAUPAR-ChIRP-32	GTACTACATACAGAAACCGAGG	odd
PAUPAR-ChIRP-33	AACCATGCGTACATCAATATTT	even
PAUPAR-ChIRP-34	ACCATGCGTACATCAATATTTG	odd
PAUPAR-ChIRP-35	CGAATGTTTGTATGCCATATTT	even
PAUPAR-ChIRP-36	AACTGACATTAACAGGGAGGTA	odd
PAUPAR-ChIRP-37	CCAGGCCCTAAAATAACCTCAA	even
PAUPAR-ChIRP-38	CTTACACTATAGGTGAATTGGG	odd
PAUPAR-ChIRP-39	AGCCTATTTGTCACAATTGTAG	even
PAUPAR-ChIRP-40	CAGCCTATTTGTCACAATTGTA	odd
PAUPAR-ChIRP-41	ATTCAAGTTCCTTGCATCAAAA	even
PAUPAR-ChIRP-42	AAATTCAAGTTCCTTGCATCAA	odd
